# Circulating hormones and risk of gastric cancer by subsite in three cohort studies

**DOI:** 10.1007/s10120-023-01414-0

**Published:** 2023-07-16

**Authors:** Harinakshi Sanikini, Carine Biessy, Sabina Rinaldi, Anne-Sophie Navionis, Audrey Gicquiau, Pekka Keski-Rahkonen, Agneta Kiss, Stephanie J. Weinstein, Demetrius Albanes, Antonio Agudo, Mazda Jenab, Elio Riboli, Marc J. Gunter, Gwen Murphy, Amanda J. Cross

**Affiliations:** 1 Department of Epidemiology and Biostatistics, School of Public Health, Imperial College London, St. Mary’s Campus, Norfolk Place, London, W2 1PG UK; 2 Section of Nutrition and Metabolism, International Agency for Research on Cancer, Lyon, France; 3 Metabolic Epidemiology Branch, Division of Cancer Epidemiology and Genetics, National Cancer Institute, Rockville, MD USA; 4 Unit of Nutrition and Cancer, Catalan Institute of Oncology-ICO, L’Hospitalet de Llobregat, Spain; 5 Nutrition and Cancer Group, Epidemiology, Public Health, Cancer Prevention and Palliative Care Program, Bellvitge Biomedical Research Institute-IDIBELL, L’Hospitalet de Llobregat, Spain; 6 Cancer Screening and Prevention Research Group, Department of Surgery and Cancer, Imperial College London, London, UK

**Keywords:** Hormones, Gastric, Cancer

## Abstract

**Background:**

Obesity has been positively associated with gastric cancer. Excess fat impacts hormones, which have been implicated in carcinogenesis. We investigated obesity-related hormones and cardia gastric cancer (CGC) and non-cardia gastric cancer (NCGC) risk.

**Methods:**

Nested case–control studies were conducted within the European Prospective Investigation into Cancer and Nutrition (EPIC) cohort (61 CGCs, and 172 NCGCs and matched controls) and the Alpha-Tocopherol, Beta-Carotene Cancer Prevention (ATBC) study (100 CGCs and 65 NCGCs and matched controls); serum hormones were measured. In UK-Biobank (*n* = 458,713), we included 137 CGCs and 92 NCGCs. Sex-specific analyses were conducted. For EPIC and ATBC, odds ratios (ORs), and for UK-Biobank hazard ratios (HRs), were estimated using conditional logistic regression and Cox regression, respectively.

**Results:**

Insulin-like growth-factor-1 was positively associated with CGC and NCGC in EPIC men (OR_per 1-SD increase_ 1.94, 95% CI 1.03–3.63; OR_per 1-SD increase_ 1.63, 95% CI 1.05–2.53, respectively), with similar findings for CGC in UK-Biobank women (HR_per 1-SD increase_ 1.76, 95% CI 1.08–2.88). Leptin in EPIC men and C-peptide in EPIC women were positively associated with NCGC (OR_T3 vs. T1_ 2.72, 95% CI 1.01–7.34 and OR_per 1-SD increase_ 2.17, 95% CI 1.19–3.97, respectively). Sex hormone-binding globulin was positively associated with CGC in UK-Biobank men (HR_per 1-SD increase_ 1.29, 95% CI 1.02–1.64). Conversely, ghrelin was inversely associated with NCGC among EPIC and ATBC men (OR_per 1-SD increase_ 0.53, 95% CI 0.34–0.84; OR_per 1-SD increase_ 0.22, 95% CI 0.10–0.50, respectively). In addition, dehydroepiandrosterone was inversely associated with CGC in EPIC and ATBC men combined.

**Conclusions:**

Some obesity-related hormones influence CGC and NCGC risk.

**Supplementary Information:**

The online version contains supplementary material available at 10.1007/s10120-023-01414-0.

## Introduction

Gastric cancer is among the most commonly diagnosed cancers globally [1]. There are striking differences in the risk factor profiles for the two gastric cancer subsites: cardia gastric cancer (CGC) and non-cardia gastric cancer (NCGC). Obesity is positively associated with CGC [2–5], and with gastro-esophageal reflux disease [6], while *Helicobacter pylori* infection is one of the most significant risk factors for NCGC [6]. Incidence rates for gastric cancer overall are two-fold higher in men than in women [1], but this sex imbalance is more pronounced in CGC than in NCGC [5]. Obesity can alter circulating hormone levels: insulin, leptin and sex steroid hormones are higher in obese individuals, while adiponectin levels are lower [7].

Insulin regulates glucose metabolism [8] and, in non-fasting blood samples, it can be estimated using C-peptide levels [9]. A previous nested case–control study showed a positive association between insulin and C-peptide levels and gastric cancer [10]. Insulin-like growth factor-1 (IGF-1), similar in structure to insulin, is regulated via insulin-like growth factor-binding proteins (IGFBPs) and 80% of IGF-1 is bound to IGFBP-3 [11]. Previous prospective studies investigated the associations between serum levels of IGF-1 and IGFBP-3 and risk of gastric cancer and reported no associations [12–14]. However, gastric cancer subsite was not previously examined.

Adiponectin and leptin are hormones produced in adipose tissue. Adiponectin regulates glucose and lipid metabolism [15] and leptin regulates appetite and energy balance [16]. Previous studies have reported lower levels of adiponectin and leptin in gastric cancer patients than controls [17, 18]. In contrast to leptin, ghrelin is produced in the stomach and stimulates appetite, increasing food intake [16]. Previous studies have reported low levels of ghrelin are associated with an increased risk of both CGC and NCGC [19, 20] but data on sex-specific associations are limited.

Sex steroid hormones influence body fat distribution [21] and adipose tissue is a major source of estrogens and androgens in obese individuals. It has been hypothesized that sex hormones may explain the male predominance in gastric cancer. Several cohort studies have investigated the associations between self-reported reproductive factors, such as hormone replacement therapy use, and gastric cancer risk [3, 4, 22, 23]. However, prospective studies examining the associations between circulating sex steroids and risk of gastric cancer by subsite are also limited [24–26].

We aimed to investigate the associations between a range of circulating hormones related to obesity and risk of gastric cancer by subsite in three prospective cohort studies.

## Methods

### Study population

The three participating prospective cohort studies were: the European Prospective Investigation into Cancer and Nutrition (EPIC) cohort [27], the Alpha-Tocopherol, Beta-Carotene Cancer Prevention (ATBC) Study [28] and the UK-Biobank cohort [29]. The study design and rationale of each participating cohort has been described in detail previously [27–29].

The EPIC cohort includes 521,324 men and women, recruited between 1992 and 2000 from 10 European countries; for this analysis, we had follow-up through 2012 [27]. The ATBC Study is a randomized controlled trial of 29,133 men initiated in 1985 to investigate the effects of alpha-tocopherol and beta-carotene supplementation on cancer risk in Finnish smokers; this analysis included data on incident cancers through 2012 [28]. The UK-Biobank cohort consists of 502,524 men and women from the UK enrolled between 2006 and 2010 to investigate a wide range of health conditions and illnesses, with follow-up through 2016 [29]. Characteristics of the participating cohorts are presented in Supplementary Table 1. Each study was approved by the relevant local institutional review boards.

Within EPIC and ATBC, we conducted nested case–control studies. In both cohorts, incidence-density sampling was used where one control was matched to each case (controls were alive and cancer-free at the time of case diagnosis). In EPIC, controls were matched to cases on recruitment center, sex, age at enrolment (± 3 years) and date (± 3 months) and time (± 3 h) of blood sample collection (Supplementary Table 1). In the ATBC study, case and control subjects were matched on age at randomization (± 1 year), and date of blood draw (± 30 days) (Supplementary Table 1). UK-Biobank was analyzed as a cohort study because circulating hormone levels were available on the majority of participants; we excluded participants with prevalent cancer at recruitment (*n* = 43,811), leaving 458,713 available for analysis.

Cancer cases were mostly identified through cancer registries. Cases were first primary incident gastric cancer coded according to the International Classification of Diseases (ICD-9 or 10). CGC and NCGC included topography codes C16.0 and C16.1–16.6, respectively. In EPIC, there were 61 CGC cases and 61 controls in men (there were too few CGC cases among women), and 172 NCGC cases and 172 controls in men and women combined. In the all-male ATBC cohort, there were 100 CGC cases matched to 100 controls and 65 NCGC cases matched to 65 controls. Of these CGC and NCGC cases and controls in ATBC, 84 CGC cases and 10 controls, and all of the 65 NCGC cases and 65 controls overlapped with a previous nested case–control study in ATBC [19]. In the UK-Biobank cohort, there were 137 CGC cases and 92 NCGC cases in men and women combined.

### Data and sample collection

All three studies collected information on socio-demographic, dietary, lifestyle and medical history mainly via a self-administered questionnaire at baseline. Furthermore, anthropometric measurements and fasting serum samples were collected at baseline. Details of data and specimen collection have been described previously by the individual studies [27–29].

### Hormone measurements

In each of the three cohorts, there were slight differences in the hormones measured due to volume availability.

*EPIC:* The hormones measured in EPIC for CGC and NCGC were insulin, IGF-1, IGFBP-3, C-peptide, adiponectin, leptin, ghrelin, androstenedione, dehydroepiandrosterone (DHEA), estrone, estradiol, sex hormone-binding globulin (SHBG), progesterone and testosterone.

*ATBC:* In ATBC, hormones measured for CGC included insulin, IGF-1, IGFBP-3, leptin and adiponectin, which were new measurements, while ghrelin, androstenedione, androsterone, DHEA, estrone, estradiol, SHBG, dihydrotestosterone and testosterone were measured as part of previous nested case–control studies [19, 24]. Furthermore, most of the hormones measured for NCGC in ATBC were new measurements including insulin, IGF-1, IGFBP-3, adiponectin, leptin, androstenedione, DHEA, estrone, estradiol, SHBG, progesterone and testosterone, while only ghrelin had been measured for a previous nested case–control study [19].

For EPIC and most of the biomarkers in ATBC mentioned above, assays were performed at the International Agency for Research on Cancer (IARC) (Lyon, France) using commercially available immunoassays: adiponectin, leptin, and insulin were measured using electrochemioluminescence assays by Meso Scale Diagnostics (Rockville, MD, USA), IGF-1 and IGFBP-3 were measured by ELISAs from R&D (Minneapolis, USA), SHBG by ELISAs from DRG Diagnostics (Marburg, Germany) and ghrelin by an ELISA kit from Merck (Fontenay sous Bois, France). In ATBC, ghrelin was measured for CGC and NCGC by radioimmunoassay using reagents obtained from Millipore Linco Research (St Charles, Missouri, USA). The intra-batch coefficients of variation (CV) values were: 5.4–7.2% for insulin, 3.4–6.4% for C-peptide, 1.8–2.4% for IGF-1, 2.1–3.3% for IGFBP-3, 11.6% for ghrelin, and 1.8–3.0% for SHBG. Sex steroids were measured for CGC and NCGC in EPIC and for NCGC in ATBC by liquid chromatography–mass spectrometry (Ultimate 3000-Q-Exactive, Thermo Scientific) at IARC using an adaptation of a previously published method [30]. In brief, serum samples were prepared by liquid–liquid extraction, derivatized by 1,2-dimethylimidazole-5-sulfonyl chloride, with separation on a reversed phase column and ionization using atmospheric pressure chemical ionization in positive polarity [30]. In ATBC, sex steroids were measured for CGC by gas chromatography–mass spectrometry performed at the Pharmacogenomics Laboratory of Laval University (Quebec, Canada). The intra-batch CV values were: 2.5–3.5% for androstenedione, 2.9–5.2% for DHEA, 5.1–7.6% for estrone, 4.5–7.2% for estradiol, 1.8–3.3% for testosterone and 3.9–7.6% for progesterone. Serum concentrations of free estradiol and free testosterone were calculated from absolute concentrations of estradiol and testosterone, respectively, and SHBG using mass action equations [31].

*UK-Biobank:* A wide range of biomarkers were already measured in all UK-Biobank participants [32]. In addition to IGF-1, we were able to examine glucose, as well as glycated hemoglobin (HbA1c), SHBG and testosterone. Furthermore, C-reactive protein (CRP), a marker of inflammation, was also examined as evidence shows that chronic inflammation is associated with obesity and may contribute to cancer development [33]. Details on assay methods have been published previously [32]. The within-laboratory CV for low, medium and high internal quality control samples for the examined biomarkers were: 5.3–6.2% for IGF-1, 1.5–1.8% for glucose, 1.7–2.3% for CRP, 5.2–5.7% for SHBG and 3.7–8.3% for testosterone [32].

### Statistical analysis

Hormone measurements were log transformed and both categorical (tertiles) and continuous (per 1-standard deviation (SD) increase) variables were analyzed. In EPIC and ATBC, categories were defined on the distribution among control subjects, whereas in UK-Biobank, it was based on the entire cohort. For hormone variables, those with missing values were assigned to a missing category. For instance, we classified hormones into categories (IGF-1 included those with values < 82, 82–104, > 104 (ng/mL) and those who were missing IGF-1 data). Also, for covariates with missing values, missing indicators were used. Baseline characteristics among cases and controls were compared using paired sampled t-tests (continuous variables) and paired Chi-squared tests (categorical variables).

For the two nested case–control studies (EPIC and ATBC), we used conditional logistic regression models to estimate odds ratio (ORs) and 95% confidence intervals (CIs) for the association between hormones and gastric cancer by subsite. Sex-specific analyses were conducted. Models were adjusted for education (EPIC: none, primary school, technical/professional, secondary school, university; ATBC: 8th grade or less, less than high school, high school graduate or general educational development, some college or technical school, college graduate), smoking (EPIC: never smoker, current smoker of 1–15, 16–25 or 26+ cigarettes/day, former smoker who stopped ≤ 10, 11–20 or 20 + years ago, current or occasional pipe/cigar, smoking unknown/missing; ATBC: < 24, 24 to < 35, 35 to < 46, ≥ 46 pack years) and body mass index (BMI, kg/m^2^, continuous). In addition, a pooled analysis of EPIC men with ATBC (all men) was performed to enhance statistical power since these two studies used the same study design (nested case–control studies); this pooled analysis did not include UK-Biobank since this was analyzed as a cohort study because, unlike EPIC and ATBC, biomarkers were measured on the entire cohort. Models were adjusted for education level, study center, smoking, and BMI. Additionally, for CGC in ATBC, we conducted a sensitivity analysis to examine the exposures measured as part of a previous nested case–control study and those that were not.

For UK-Biobank, hazard ratios (HRs) and 95% CIs were computed using Cox proportional hazard regression models, with age as the primary time variable. Entry time was age at recruitment and exit time was age at diagnosis, death or last date at which follow-up was considered complete. Sex-specific analyses were conducted. Models were stratified by age at recruitment in 5-year categories, Townsend deprivation index (quintiles) and recruitment center. Mean and SD or frequencies were computed for baseline characteristics. Models were adjusted for education (none; CSEs/O levels/GCSEs or equivalent; vocational qualifications {NVQ/HND/HNC, A-levels/AS levels or equivalent}; other qualifications; college/university degree; unknown), smoking (never, former and current) and BMI (kg/m^2^, continuous).

Linear trend tests were conducted for hormones by assigning the median value to each category as a continuous variable in the models. Pearson correlations were computed between hormones in each study. We also examined models mutually adjusted for (1) all of the gut/adipokines and (2) all of the sex hormones. A sensitivity analysis was performed by excluding gastric cancer cases diagnosed in the first year of follow-up. Furthermore, for hormones without sex differences in EPIC and UK-Biobank, we conducted combined sex analyses. All analyses were conducted using SAS 9.4 software (SAS Institute, Cary, NC) and *P* values < 0.05 were considered statistically significant.

## Results

The median time between blood collection and cancer diagnosis in EPIC was 8.0 years for CGC and 8.4 years for NCGC, in ATBC, it was 9.0 years for CGC and 7.0 years for NCGC, and in UK-Biobank, it was 2.8 years for CGC and 3.2 years for NCGC (Supplementary Table 2, 3 and 4, respectively). In EPIC, CGC cases had higher red meat intake than controls (*P* = 0.04); while NCGC cases were less educated (*P* = 0.006) and had lower levels of IGFBP-3 (*P* = 0.02) and ghrelin (*P* = 0.009) than controls (Supplementary Table 2). In ATBC, CGC cases had higher BMI (*P* = 0.02) and lower levels of adiponectin (*P* = 0.03) and ghrelin (*P* = 0.02) than controls, while NCGC cases had lower vegetable intake (*P* = 0.03) and lower levels of insulin (*P* = 0.02), and ghrelin (*P* < 0.0001) compared to controls (Supplementary Table 3). In UK-Biobank, CGC cases had a lower education level (*P* = 0.0002), were older (*P* < 0.0001), had higher BMI (*P* < 0.0001), and were more likely to be smokers (*P* < 0.0001) and alcohol consumers (*P* < 0.0001); in addition, they had higher levels of glucose (*P* = 0.007), HbA1c (*P* = 0.02), CRP (*P* = 0.008), and testosterone (*P* < 0.0001), but lower levels of SHBG (*P* = 0.002) than non-cases (Supplementary Table 4). For NCGC, cases were also less educated (*P* = 0.001), older (*P* < 0.0001), consumed less alcohol (*P* = 0.02) and had higher levels of glucose (*P* = 0.04), HbA1c (*P* = 0.001), CRP (*P* = 0.009) and testosterone (*P* = 0.006) but had lower levels of IGF-1 (*P* = 0.008) compared to non-cases (Supplementary Table 4).

In EPIC men, IGF-1 was positively associated with CGC and NCGC (per 1-SD increase: adjusted OR 1.94, 95% CI 1.03–3.63 and 1.63, 95% CI 1.05–2.53, respectively) (Table 1). In addition, leptin was also positively associated with NCGC but only in categorical data (OR 2.72, 95% CI 1.01–7.34 for the highest vs. lowest category), whereas ghrelin was inversely associated with NCGC (adjusted OR 0.30, 95% CI 0.11–0.87 for the highest vs. lowest category; OR 0.53, 95% CI 0.34–0.84 per 1-SD increase) (Table 1). Among EPIC women, C-peptide was positively associated with NCGC (adjusted OR 3.84, 95% CI 1.02–14.43 for the highest vs. lowest category and OR 2.17, 95% CI 1.19–3.97 per 1-SD increase) (Table 2); however, there were no other associations with CGC or NCGC. Furthermore, in sex-combined analyses (Supplementary table 5), we found IGFBP-3 and ghrelin were inversely associated with NCGC in EPIC (adjusted OR 0.70, 95% CI 0.51–0.95 and OR 0.66, 95% CI 0.49–0.89 per 1-SD increase, respectively), while leptin was positively associated with NCGC (adjusted OR 1.60, 95% CI 1.06–2.42 per 1-SD increase).

**Table 1:** Odds ratios and 95% confidence intervals for circulating hormones and gastric cancer by subsite in men from the EPIC study

	CGC			NCGC		
	Cases/controls (61/61)	Crude OR^a^ (95% CI)	Adjusted OR^b^ (95% CI)	Cases/controls (104/104)	Crude OR^a^ (95% CI)	Adjusted OR^b^ (95% CI)
Insulin (pg/mL)^c^						
< 243	5/5	Reference		14/15	Reference	Reference
243–333	4/6	0.88 (0.21–3.65)	–	8/15	0.61 (0.20–1.91)	0.98 (0.22–4.47)
> 333	7/5	2.72 (0.22–33.0)	–	23/15	1.58 (0.58–4.30)	1.31 (0.30–5.67)
*P trend*		0.63			0.20	0.91
Per 1-SD increase		1.07 (0.38–2.98)	1.70 (0.16–18.50)		1.26 (0.83–1.92)	1.21 (0.61–2.40)
C-peptide (pmol/L)^d^						
< 552	19/16	Reference	Reference	34/37	Reference	Reference
552–968	21/22	0.79 (0.31–2.02)	0.99 (0.30–3.26)	33/33	1.09 (0.56–2.14)	1.03 (0.45–2.33)
> 968	21/23	0.75 (0.30–1.90)	0.66 (0.23–1.91)	35/32	1.20 (0.61–2.36)	1.28 (0.55–3.01)
*P trend*		0.82	0.67		0.87	0.82
Per 1-SD increase		1.11 (0.76–1.62)	0.99 (0.65–1.53)		1.02 (0.79–1.32)	1.03 (0.75–1.43)
IGF-1 (ng/mL)						
< 82	15/18	Reference	Reference	28/36	Reference	Reference
82–104	20/19	1.33 (0.52–3.41)	1.98 (0.59–6.62)	38/37	1.32 (0.68–2.56)	1.63 (0.75–3.51)
> 104	26/24	1.51 (0.49–4.71)	2.84 (0.64–12.52)	38/31	1.85 (0.82–4.17)	2.67 (0.97–7.31)
*P trend*		0.76	0.37		0.33	0.15
Per 1-SD increase		1.50 (0.95–2.39)	1.94 (1.03–3.63)		1.37 (0.97–1.94)	1.63 (1.05–2.53)
IGFBP-3 (ng/mL)						
< 1939	28/22	Reference	Reference	49/33	Reference	Reference
1939–2265	11/21	0.40 (0.15–1.07)	0.40 (0.11–1.41)	23/33	0.49 (0.25–0.96)	0.49 (0.23–1.07)
> 2265	22/18	0.94 (0.37–2.37)	1.67 (0.47–5.98)	32/38	0.55 (0.27–1.08)	0.65 (0.29–1.42)
*P trend*		0.14	0.13		0.08	0.18
Per 1-SD increase		0.92 (0.63–1.33)	1.09 (0.69–1.73)		0.68 (0.48–0.96)	0.73 (0.50–1.07)
Adiponectin (ng/mL)						
< 5057	17/18	Reference	Reference	44/37	Reference	Reference
5057–7023	19/18	1.15 (0.41–3.22)	0.92 (0.24–3.51)	28/36	0.62 (0.30–1.26)	0.83 (0.36–1.92)
> 7023	25/25	1.08 (0.44–2.66)	1.13 (0.37–3.42)	32/31	0.84 (0.41–1.72)	1.16 (0.47–2.81)
*P trend*		0.97	0.93		0.41	0.76
Per 1-SD increase		0.96 (0.69–1.34)	1.03 (0.68–1.56)		0.91 (0.68–1.23)	1.06 (0.73–1.54)
Leptin (pg/mL)						
< 2142	14/13	Reference	Reference	25/40	Reference	Reference
2142–4105	28/24	1.07 (0.43–2.68)	0.83 (0.23–2.98)	41/31	2.43 (1.12–5.24)	2.99 (1.17–7.64)
> 4105	17/22	0.70 (0.25–1.92)	0.42 (0.09–1.97)	37/32	1.97 (0.96–4.04)	2.72 (1.01–7.34)
*P trend*		0.60	0.47		0.06	0.06
Per 1-SD increase		0.88 (0.62–1.24)	0.73 (0.42–1.29)		1.25 (0.94–1.67)	1.39 (0.91–2.12)
Ghrelin (pg/mL)						
< 173	23/19	Reference	Reference	49/36	Reference	Reference
173–276	14/17	0.72 (0.30–1.72)	0.60 (0.18–2.05)	33/37	0.52 (0.23–1.15)	0.40 (0.15–1.04)
> 276	24/25	0.82 (0.38–1.80)	0.62 (0.23–1.71)	22/31	0.39 (0.17–0.93)	0.30 (0.11–0.87)
*P trend*		0.74	0.59		0.08	0.05
Per 1-SD increase		0.86 (0.61–1.20)	0.75 (0.48–1.16)		0.57 (0.39–0.85)	0.53 (0.34–0.84)
Androstenedione (pmol/L)						
< 2291	15/16	Reference	Reference	25/25	Reference	Reference
2291–3203	15/12	1.70 (0.36–7.91)	0.97 (0.05–18.41)	28/29	0.97 (0.45–2.11)	1.16 (0.45–3.04)
> 3203	15/17	1.08 (0.27–4.26)	0.55 (0.04–7.06)	25/24	1.06 (0.44–2.56)	1.28 (0.36–4.55)
*P trend*		0.71	0.79		0.98	0.92
Per 1-SD increase		1.41 (0.80–2.49)	1.55 (0.67–3.58)		0.93 (0.64–1.36)	1.07 (0.62–1.86)
DHEA (pmol/L)						
< 5540	19/18	Reference	Reference	25/22	Reference	Reference
5540–8496	11/12	0.84 (0.27–2.65)	0.30 (0.04–2.17)	25/29	0.63 (0.22–1.80)	0.82 (0.24–2.77)
> 8496	15/15	0.89 (0.24–3.35)	0.46 (0.05–4.15)	28/27	0.77 (0.27–2.15)	0.97 (0.27–3.52)
*P trend*		0.96	0.49		0.68	0.91
Per 1-SD increase		1.19 (0.71–1.99)	1.06 (0.50–2.26)		1.02 (0.68–1.54)	1.12 (0.63–1.98)
Estrone (pmol/L)						
< 102	15/12	Reference	Reference	28/29	Reference	Reference
102–136	14/17	0.61 (0.19–1.91)	0.56 (0.10–3.12)	28/23	1.22 (0.59–2.51)	1.46 (0.56–3.78)
> 136	16/16	0.74 (0.23–2.37)	0.69 (0.13–3.61)	22/26	0.81 (0.34–1.94)	0.65 (0.21–1.99)
*P trend*		0.70	0.80		0.64	0.37
Per 1-SD increase		1.10 (0.73–1.65)	1.10 (0.59–2.05)		0.99 (0.69–1.43)	0.79 (0.49–1.27)
Estradiol (pmol/L)						
< 52.5	11/10	Reference	Reference	20/30	Reference	Reference
52.5–72.7	17/19	0.80 (0.25–2.55)	0.30 (0.04–2.23)	32/23	2.49 (1.01–6.15)	3.09 (0.98–9.73)
> 72.7	17/16	0.96 (0.27–3.37)	0.50 (0.06–4.40)	26/25	1.61 (0.73–3.57)	1.07 (0.40–2.83)
*P trend*		0.90	0.47		0.14	0.12
Per 1-SD increase		1.04 (0.68–1.59)	0.90 (0.42–1.94)		1.28 (0.94–1.74)	1.11 (0.74–1.66)
Free estradiol (pmol/L)						
< 1.4	9/11	Reference	Reference	20/30	Reference	Reference
1.4–1.8	22/16	1.63 (0.55–4.79)	1.89 (0.39–9.12)	25/25	1.50 (0.65–3.48)	0.99 (0.34–2.92)
> 1.8	14/18	0.96 (0.31–2.94)	0.70 (0.10–4.85)	33/23	2.13 (0.97–4.69)	1.65 (0.59–4.61)
*P trend*		0.48	0.40		0.17	0.55
Per 1-SD increase		0.95 (0.63–1.43)	0.87 (0.41–1.88)		1.34 (0.96–1.87)	1.16 (0.74–1.83)
SHBG (nmol/L)^e^						
< 29.4	11/17	Reference	Reference	29/38	Reference	Reference
29.4–46.3	24/24	1.55 (0.62–3.92)	1.01 (0.32–3.19)	41/31	1.78 (0.88–3.60)	1.99 (0.86–4.62)
> 46.3	26/20	2.09 (0.77–5.70)	1.80 (0.51–6.39)	34/35	1.36 (0.63–2.92)	2.26 (0.75–6.78)
*P trend*		0.35	0.54		0.27	0.22
Per 1-SD increase		1.22 (0.82–1.82)	1.15 (0.69–1.92)		1.00 (0.74–1.34)	1.13 (0.77–1.66)
Testosterone (pmol/L)						
< 11,207	13/15	Reference	Reference	25/25	Reference	Reference
11,207–14,606	15/18	0.99 (0.40–2.47)	1.08 (0.28–4.20)	21/24	0.81 (0.34–1.93)	0.62 (0.18–2.12)
> 14,606	17/12	1.70 (0.58–4.98)	1.23 (0.24–6.40)	32/29	1.13 (0.55–2.34)	1.13 (0.42–3.03)
*P trend*		0.53	0.97		0.78	0.62
Per 1-SD increase		1.25 (0.80–1.94)	0.94 (0.49–1.80)		1.07 (0.80–1.44)	1.13 (0.74–1.74)
Free testosterone (pmol/L)						
< 205	21/19	Reference	Reference	18/22	Reference	Reference
205–258	10/13	0.71 (0.26–1.93)	0.46 (0.10–2.15)	30/28	1.38 (0.58–3.27)	1.57 (0.51–4.85)
> 258	14/13	1.05 (0.34–3.27)	0.42 (0.06–2.82)	30/28	1.43 (0.56–3.68)	1.73 (0.50–6.07)
*P trend*		0.77	0.50		0.72	0.66
Per 1-SD increase		1.05 (0.68–1.64)	0.87 (0.45–1.71)		1.20 (0.81–1.75)	1.27 (0.74–2.17)
Progesterone (pmol/L)						
< 122	16/15	Reference	Reference	27/26	Reference	Reference
122–186	10/16	0.53 (01.6–1.82)	0.63 (0.09–4.57)	26/24	1.04 (0.48–2.25)	1.68 (0.61–4.64)
> 186	19/14	1.33 (0.41–4.29)	2.60 (0.31–21.46)	25/28	0.84 (0.37–1.89)	1.46 (0.46–4.60)
*P trend*		0.31	0.25		0.86	0.60
Per 1-SD increase		1.14 (0.75–1.72)	1.38 (0.68–2.80)		1.05 (0.72–1.52)	1.33 (0.77–2.30)

^a^Crude model was integrally adjusted for the matching factors, including study center, sex, age at recruitment and date/time of blood collection

^b^Adjusted model was based on crude model with further adjustment for education level, smoking, and body mass index

^c^For insulin, only fasting subjects were included

^d^Additionally adjusted for fasting status

^e^Additionally adjusted for insulin

All variables were log transformed

Not all of the cases/controls will sum to the total due to missing data

*CGC* cardia gastric cancer, *CI* confidence interval, *DHEA* dehydroepiandrosterone, *IGF-1* insulin-like growth factor-1, *IGFBP-3* insulin-like growth factor-binding protein-3, *NCGC* non-cardia gastric cancer, *OR* odds ratio, *SD* standard deviation, *SHBG* sex hormone-binding globulin

**Table 2:** Odds ratios and 95% confidence intervals for circulating hormones and gastric non-cardia cancer in women from the EPIC study

	NCGC		
	Cases/controls (68/68)	Crude OR^a^ (95% CI)	Adjusted OR^b^ (95% CI)
Insulin (pg/mL)^c^			
< 202	8/10	Reference	Reference
202–295	10/13	0.93 (0.27–3.24)	1.38 (0.27–7.09)
> 295	14/9	1.77 (0.54–5.81)	5.20 (0.85–31.85)
*P trend*		0.49	0.14
Per 1-SD increase		1.18 (0.72–1.94)	2.19 (0.84–5.68)
C-peptide (pmol/L)^d^			
< 500	16/23	Reference	Reference
500–753	19/25	1.02 (0.43–2.43)	1.16 (0.37–3.69)
> 753	33/20	3.20 (1.17–8.78)	3.84 (1.02–14.43)
*P trend*		0.05	0.08
Per 1-SD increase		1.90 (1.22–2.95)	2.17 (1.19–3.97)
IGF-1 (ng/mL)			
< 66.8	27/24	Reference	Reference
66.8–84.8	27/21	1.21 (0.49–2.99)	1.84 (0.58–5.85)
> 84.8	14/23	0.39 (0.13–1.17)	0.76 (0.19–2.94)
*P trend*		0.13	0.39
Per 1-SD increase		0.72 (0.46–1.14)	1.01 (0.56–1.81)
IGFBP-3 (ng/mL)			
< 2175	25/22	Reference	Reference
2175–2500	27/21	0.89 (0.32–2.47)	1.33 (0.38–4.68)
> 2500	16/25	0.43 (0.14–1.36)	0.60 (0.14–2.61)
*P trend*		0.21	0.38
Per 1-SD increase		0.71 (0.47–1.06)	0.86 (0.51–1.45)
Adiponectin (ng/mL)			
< 8479	21/22	Reference	Reference
8479–13,988	33/20	2.02 (0.80–5.15)	1.85 (0.57–5.96)
> 13,988	14/26	0.61 (0.25–1.50)	0.60 (0.19–1.89)
*P trend*		0.04	0.14
Per 1-SD increase		0.86 (0.62–1.19)	0.84 (0.55–1.29)
Leptin (pg/mL)			
< 6870	18/21	Reference	Reference
6870–15,472	20/21	1.14 (0.45–2.85)	2.03 (0.51–8.01)
> 15,472	30/26	1.55 (0.58–4.17)	3.73 (0.76–18.28)
*P trend*		0.67	0.26
Per 1-SD increase		1.19 (0.80–1.77)	1.66 (0.88–3.16)
Ghrelin (pg/mL)			
< 193	25/20	Reference	Reference
193–345	24/22	0.87 (0.39–1.96)	1.03 (0.38–2.83)
> 345	19/26	0.59 (0.25–1.36)	0.52 (0.18–1.52)
*P trend*		0.44	0.35
Per 1-SD increase		0.79 (0.56–1.11)	0.79 (0.51–1.22)
Androstenedione (pmol/L)			
< 1378	15/15	Reference	Reference
1378–2165	25/19	1.07 (0.38–2.99)	1.01 (0.28–3.63)
> 2165	13/19	0.63 (0.19–2.07)	1.20 (0.23–6.28)
*P trend*		0.61	0.97
Per 1-SD increase		0.88 (0.55–1.39)	1.21 (0.65–2.24)
DHEA (pmol/L)			
< 4024	16/18	Reference	Reference
4024–8310	24/16	1.69 (0.58–4.87)	2.26 (0.58–8.86)
> 8310	13/19	0.72 (0.21–2.47)	1.54 (0.29–8.30)
*P trend*		0.25	0.48
Per 1-SD increase		0.81 (0.50–1.31)	1.07 (0.58–1.98)
Estrone (pmol/L)			
< 53.6	16/17	Reference	Reference
53.6–83.2	22/17	1.35 (0.54–3.36)	2.20 (0.63–7.67)
> 83.2	15/19	0.79 (0.28–2.18)	1.75 (0.41–7.56)
*P trend*		0.55	0.47
Per 1-SD increase		1.12 (0.74–1.70)	1.49 (0.88–2.52)
Estradiol (pmol/L)			
< 9.2	14/16	Reference	Reference
9.2–16.1	20/14	1.82 (0.64–5.13)	6.45 (1.09–38.40)
> 16.1	13/17	0.74 (0.25–2.18)	2.47 (0.29–21.04)
*P trend*		0.33	0.12
Per 1-SD increase		0.99 (0.64–1.53)	1.12 (0.59–2.15)
Free estradiol (pmol/L)			
< 0.2	16/13	Reference	Reference
0.2–0.4	17/17	0.83 (0.31–2.25)	1.82 (0.41–8.00)
> 0.4	14/17	0.67 (0.24–1.87)	1.36 (0.23–8.26)
*P trend*		0.74	0.73
Per 1-SD increase		1.03 (0.67–1.59)	1.13 (0.59–2.16)
SHBG (nmol/L)^e^			
< 44.6	23/21	Reference	Reference
44.6–63.7	27/25	0.97 (0.41–2.33)	1.44 (0.45–4.62)
> 63.7	18/22	0.75 (0.31–1.81)	0.91 (0.26–3.15)
*P trend*		0.77	0.67
Per 1-SD increase		0.82 (0.57–1.17)	0.81 (0.48–1.36)
Testosterone (pmol/L)			
< 454	21/16	Reference	Reference
454–697	18/18	0.78 (0.30–2.00)	0.86 (0.26–2.82)
> 697	14/19	0.58 (0.23–1.47)	0.49 (0.13–1.94)
*P trend*		0.51	0.59
Per 1-SD increase		0.91 (0.62–1.32)	1.01 (0.62–1.65)
Free testosterone (pmol/L)			
< 5.4	16/16	Reference	Reference
5.4–8.5	21/18	1.24 (0.40–3.85)	2.05 (0.43–9.73)
> 8.5	16/19	0.85 (0.29–2.48)	1.20 (0.23–6.30)
*P trend*		0.76	0.62
Per 1-SD increase		0.97 (0.66–1.43)	1.07 (0.64–1.78)
Progesterone (pmol/L)			
< 71.5	15/16	Reference	Reference
71.5–101	19/20	1.02 (0.38–2.79)	0.83 (0.23–3.06)
> 101	19/17	1.22 (0.44–3.40)	1.41 (0.39–5.11)
*P trend*		0.91	0.71
Per 1-SD increase		1.11 (0.77–1.61)	1.12 (0.67–1.88)

All variables were log transformed

Not all of the cases/controls will sum to the total due to missing data

*CI* confidence interval, *DHEA* dehydroepiandrosterone, *IGF-1* insulin-like growth factor-1, *IGFBP-3* insulin-like growth factor-binding protein-3, *NCGC* non-cardia gastric cancer, *OR* odds ratio, *SD* standard deviation, *SHBG* sex hormone-binding globulin

^a^Crude model was integrally adjusted for the matching factors, including study center, sex, age at recruitment and date/time of blood collection

^b^Adjusted model was based on crude model with further adjustment for education level, smoking and body mass index

^c^For insulin, only fasting subjects were included

^d^Additionally adjusted for fasting status

^e^Additionally adjusted for insulin

In ATBC, there were no associations between circulating hormones and CGC (Table 3). For NCGC, inverse associations were observed with ghrelin, which is consistent with the findings in EPIC, and insulin (per 1-SD increase: adjusted OR 0.22, 95% CI 0.10–0.50 and OR 0.45, 95% CI 0.22–0.91, respectively) (Table 3). There were no associations for any of the sex hormones in relation to CGC or NCGC in ATBC. Furthermore, in ATBC, sensitivity analyses found no significant difference in the association for those exposures that overlapped with the previous case–control study [19] and those that did not (data not shown).

**Table 3:** Odds ratios and 95% confidence intervals for circulating hormones and gastric cancer by subsite in men from the ATBC study

	CGC			NCGC		
	Cases/controls (100/100)	Crude OR^a^ (95% CI)	Adjusted OR^b^ (95% CI)	Cases/Controls (65/65)	Crude OR^a^ (95% CI)	Adjusted OR^b^ (95% CI)
Insulin (pg/mL)						
< 184	35/41	Reference	Reference	18/12	Reference	Reference
184–325	34/33	1.21 (0.63–2.31)	0.95 (0.46–1.96)	28/21	0.91 (0.38–2.22)	0.90 (0.31–2.61)
> 325	30/25	1.39 (0.70–2.74)	0.71 (0.29–1.74)	17/30	0.41 (0.16–1.03)	0.30 (0.08–1.12)
*P trend*		0.64	0.72		0.09	0.08
Per 1-SD increase		1.10 (0.85–1.42)	0.85 (0.60–1.20)		0.63 (0.41–0.97)	0.45 (0.22–0.91)
IGF-1 (ng/mL)						
< 84.3	34/32	Reference	Reference	22/21	Reference	Reference
84.3–108	41/31	1.30 (0.69–2.46)	1.19 (0.59–2.37)	21/23	0.86 (0.36–2.08)	0.90 (0.35–2.29)
> 108	24/36	0.58 (0.28–1.19)	0.60 (0.27–1.30)	19/18	1.01 (0.39–2.58)	1.10 (0.40–3.02)
*P trend*		0.12	0.25		0.93	0.93
Per 1-SD increase		0.85 (0.64–1.13)	0.83 (0.61–1.14)		1.09 (0.73–1.64)	1.11 (0.72–1.73)
IGFBP-3 (ng/mL)						
< 1642	42/38	Reference	Reference	15/14	Reference	Reference
1642–2084	26/31	0.79 (0.41–1.49)	1.09 (0.53–2.22)	23/21	1.01 (0.40–2.56)	1.08 (0.40–2.91)
> 2084	29/28	0.94 (0.48–1.83)	1.14 (0.53–2.44)	23/26	0.79 (0.29–2.13)	0.87 (0.30–2.54)
*P trend*		0.75	0.17		0.84	0.90
Per 1-SD increase		1.01 (0.76–1.33)	1.10 (0.80–1.51)		1.07 (0.69–1.66)	1.14 (0.71–1.83)
Adiponectin (ng/mL)						
< 6065	45/23	Reference	Reference	21/29	Reference	Reference
6065–8855	27/41	0.41 (0.21–0.79)	0.41 (0.20–0.87)	24/14	2.88 (1.05–7.96)	2.58 (0.86–7.72)
> 8855	27/35	0.44 (0.21–0.89)	0.56 (0.25–1.25)	17/19	1.52 (0.60–3.85)	1.42 (0.48–4.14)
*P trend*		0.02	0.06		0.12	0.22
Per 1-SD increase		0.73 (0.55–0.96)	0.85 (0.62–1.17)		1.11 (0.78–1.57)	1.06 (0.72–1.57)
Leptin (pg/mL)						
< 1398	22/37	Reference	Reference	17/17	Reference	Reference
1398–3955	51/34	2.53 (1.24–5.16)	1.51 (0.66–3.43)	23/20	1.15 (0.51–2.61)	1.35 (0.53–3.42)
> 3955	26/28	1.62 (0.73–3.57)	0.67 (0.23–2.02)	23/26	0.87 (0.38–1.96)	1.10 (0.33–3.65)
*P trend*		0.04	0.13		0.81	0.81
Per 1-SD increase		1.21 (0.89–1.63)	0.83 (0.53–1.29)		0.75 (0.53–1.07)	0.56 (0.29–1.09)
Ghrelin (pg/mL)						
< 596	51/3	Reference	Reference	45/21	Reference	Reference
596–833	23/3	1.89 (0.08–44.94)	-	13/22	0.37 (0.16–0.88)	0.29 (0.11–0.81)
> 833	10/4	0.53 (0.02–12.54)	-	7/22	< 0.001 (< 0.001- > 999)	- < 0.001 (< 0.001- > 999)
*P trend*		0.60			0.08	0.06
Per 1-SD increase		0.45 (0.10–2.08)			0.27 (0.14–0.53)	0.22 (0.10–0.50)
Androstenedione (pmol/L)						
< 3702	23/23	Reference	Reference	30/31	Reference	Reference
3702–5084	38/34	1.17 (0.54–2.55)	1.23 (0.51–2.94)	25/21	1.26 (0.53–2.97)	1.31 (0.51–3.36)
> 5084	39/43	0.92 (0.47–1.82)	0.95 (0.43–2.11)	10/13	0.79 (0.27–2.29)	0.71 (0.21–2.37)
*P trend*		0.79	0.79		0.67	0.58
Per 1-SD increase		0.99 (0.74–1.33)	1.00 (0.71–1.40)		1.04 (0.74–1.48)	0.99 (0.69–1.44)
Androsterone (pmol/L)						
< 755	24/28	Reference	Reference		NM	NM
755–1008	26/28	1.13 (0.49–2.57)	1.24 (0.49–3.15)			
> 1008	34/28	1.50 (0.67–3.36)	2.15 (0.82–5.68)			
*P trend*		0.59	0.26			
Per 1-SD increase		1.17 (0.84–1.63)	1.25 (0.86–1.81)			
DHEA (pmol/L)						
< 7722	33/23	Reference	Reference	28/28	Reference	Reference
7722–11,836	37/34	0.72 (0.35–1.46)	0.95 (0.43–2.10)	21/18	1.11 (0.51–2.42)	1.07 (0.46–2.52)
> 11,836	29/42	0.43 (0.20–0.95)	0.51 (0.22–1.21)	7/10	0.61 (0.17–2.22)	0.68 (0.16–2.89)
*P trend*		0.10	0.03		0.65	0.82
Per 1-SD increase		0.73 (0.53–1.00)	0.75 (0.53–1.07)		0.78 (0.52–1.17)	0.73 (0.45–1.17)
Estrone (pmol/L)						
< 124	41/38	Reference	Reference	23/17	Reference	Reference
124–161	22/28	0.72 (0.35–1.49)	0.57 (0.25–1.31)	22/26	0.58 (0.23–1.48)	0.55 (0.20–1.54)
> 161	37/34	1.01 (0.53–1.93)	0.65 (0.31–1.38)	20/22	0.69 (0.31–1.55)	0.73 (0.31–1.73)
*P trend*		0.61	0.36		0.49	0.52
Per 1-SD increase		0.99 (0.72–1.36)	0.85 (0.59–1.20)		0.90 (0.67–1.20)	0.91 (0.66–1.24)
Estradiol (pmol/L)						
< 66.2	30/27	Reference	Reference	30/28	Reference	Reference
66.2–92.3	33/34	0.84 (0.38–1.86)	0.67 (0.27–1.66)	25/20	1.30 (0.53–3.19)	1.15 (0.44–3.03)
> 92.3	37/39	0.84 (0.42–1.68)	0.62 (0.29–1.34)	10/17	0.62 (0.26–1.48)	0.66 (0.26–1.70)
*P trend*		0.88	0.47		0.33	0.57
Per 1-SD increase		1.00 (0.75–1.34)	0.89 (0.65–1.23)		0.91 (0.67–1.23)	0.91 (0.66–1.26)
Free estradiol (pmol/L)						
< 1.4	27/30	Reference	Reference	26/25	Reference	Reference
1.4–1.9	41/35	1.37 (0.64–2.95)	1.09 (0.47–2.54)	22/19	1.12 (0.50–2.50)	1.25 (0.53–2.95)
> 1.9	32/35	1.07 (0.52–2.19)	0.69 (0.30–1.57)	17/21	0.79 (0.35–1.79)	0.85 (0.34–2.13)
*P trend*		0.66	0.42		0.73	0.74
Per 1-SD increase		1.05 (0.78–1.40)	0.83 (0.60–1.17)		0.93 (0.69–1.25)	0.94 (0.68–1.29)
SHBG (nmol/L)^c^						
< 49.0	31/24	Reference	Reference	31/31	Reference	Reference
49.0–75.5	33/35	0.72 (0.33–1.56)	0.89 (0.35–2.25)	21/19	1.08 (0.46–2.52)	0.87 (0.28–2.73)
> 75.5	36/41	0.65 (0.31–1.38)	0.99 (0.40–2.49)	13/15	0.84 (0.29–2.43)	0.72 (0.20–2.62)
*P trend*		0.51	0.96		0.88	0.88
Per 1-SD increase		0.91 (0.67–1.25)	1.13 (0.75–1.70)		0.96 (0.66–1.40)	0.85 (0.52–1.40)
Testosterone (pmol/L)						
< 15,075	25/20	Reference	Reference	26/32	Reference	Reference
15,075–22,877	38/35	0.89 (0.43–1.84)	1.33 (0.56–3.15)	26/17	1.70 (0.78–3.72)	1.79 (0.74–4.34)
> 22,877	36/44	0.65 (0.31–1.35)	1.06 (0.43–2.63)	6/9	0.90 (0.26–3.11)	0.86 (0.22–3.35)
*P trend*		0.46	0.75		0.30	0.30
Per 1-SD increase		0.93 (0.70–1.24)	1.02 (0.73–1.43)		1.25 (0.82–1.90)	1.19 (0.73–1.93)
Free testosterone (pmol/L)						
< 215	31/26	Reference	Reference	25/25	Reference	Reference
215–282	34/31	0.95 (0.47–1.91)	1.00 (0.47–2.14)	23/23	1.00 (0.46–2.20)	0.81 (0.33–2.03)
> 282	34/42	0.65 (0.32–1.33)	0.65 (0.30–1.42)	10/10	1.00 (0.34–2.92)	1.29 (0.36–4.65)
*P trend*		0.45	0.47		1.00	0.81
Per 1-SD increase		0.97 (0.72–1.31)	0.94 (0.68–1.30)		1.15 (0.81–1.64)	1.13 (0.75–1.71)
Dihydrotestosterone (pmol/L)						
< 1295	33/33	Reference	Reference		NM	NM
1295–1797	28/32	0.88 (0.44–1.79)	1.29 (0.55–3.05)			
> 1797	38/34	1.11 (0.56–2.18)	1.72 (0.72–4.06)			
*P trend*		0.80	0.45			
Per 1-SD increase		1.09 (0.82–1.45)	1.33 (0.91–1.95)			
Progesterone (pmol/L)						
< 146		NM	NM	23/21	Reference	Reference
146–223				27/22	1.08 (0.48–2.47)	1.15 (0.44–2.97)
> 223				15/22	0.64 (0.26–1.56)	0.72 (0.26–1.97)
*P trend*					0.43	0.57
Per 1-SD increase					0.92 (0.65–1.30)	0.94 (0.64–1.38)

All variables were log transformed

Not all of the cases/controls will sum to the total due to missing data

*CGC* cardia gastric cancer, *CI* confidence interval, *DHEA* dehydroepiandrosterone, *IGF-1* insulin-like growth factor-1, *IGFBP-3* insulin-like growth factor-binding protein-3, *NCGC* non-cardia gastric cancer, *NM* not measured, *OR* odds ratio, *SD* standard deviation, *SHBG* sex hormone-binding globulin

^a^Crude model was integrally adjusted for the matching factors, including age at randomization and date of blood draw

^b^Adjusted model was based on crude model with further adjustment for education level, smoking, and body mass index

^c^ Additionally adjusted for insulin

In pooled analyses of data from men in EPIC and ATBC (Table 4), we found an inverse association between DHEA and CGC (adjusted OR 0.45, 95% CI 0.21–0.95 for the highest vs. lowest category). In addition, ghrelin was inversely associated with NCGC (adjusted OR 0.14, 95% CI 0.05–0.41 for the highest vs. lowest category and OR 0.27, 95% CI 0.16–0.47 per 1-SD increase).

**Table 4:** Odds ratios and 95% confidence intervals for circulating hormones and gastric cancer by subsite in men from the EPIC and ATBC study

	CGC			NCGC		
	Cases/controls (161/161)	Crude OR^a^ (95% CI)	Adjusted OR^b^ (95% CI)	Cases/controls (169/169)	Crude OR^a^ (95% CI)	Adjusted OR^b^ (95% CI)
Insulin (pg/mL)						
< 204	48/44	Reference	Reference	31/29	Reference	Reference
204–328	30/43	0.64 (0.33–1.23)	0.51 (0.24–1.06)	38/33	1.12 (0.58–2.17)	1.20 (0.56–2.61)
> 328	37/28	1.17 (0.60–2.29)	0.65 (0.27–1.57)	39/46	0.81 (0.43–1.51)	0.72 (0.31–1.66)
*P trend*		0.18	0.20		0.58	0.37
Per 1-SD increase		1.09 (0.85–1.40)	0.91 (0.68–1.23)		0.87 (0.64–1.17)	0.75 (0.49–1.17)
IGF-1 (ng/mL)						
< 82.3	49/47	Reference	Reference	48/59	Reference	Reference
82.3–106	62/52	1.13 (0.67–1.90)	1.15 (0.66–1.99)	59/59	1.29 (0.73–2.27)	1.38 (0.75–2.53)
> 106	49/61	0.71 (0.39–1.28)	0.63 (0.34–1.19)	59/48	1.76 (0.93–3.32)	1.84 (0.91–3.75)
*P trend*		0.27	0.15		0.22	0.23
Per 1-SD increase		1.01 (0.80–1.28)	0.98 (0.77–1.25)		1.26 (0.97–1.63)	1.27 (0.95–1.70)
IGFBP-3 (ng/mL)						
< 1781	72/65	Reference	Reference	52/41	Reference	Reference
1781–2201	45/54	0.73 (0.42–1.27)	0.80 (0.45–1.42)	57/55	0.79 (0.45–1.40)	0.85 (0.46–1.58)
> 2201	41/39	0.91 (0.50–1.66)	0.78 (0.41–1.50)	56/69	0.60 (0.33–1.07)	0.59 (0.31–1.12)
*P trend*		0.52	0.67		0.22	0.25
Per 1-SD increase		0.98 (0.78–1.22)	0.96 (0.75–1.21)		0.81 (0.62–1.06)	0.82 (0.61–1.10)
Adiponectin (ng/mL)						
< 5457	55/34	Reference	Reference	69/74	Reference	Reference
5457–7760	44/59	0.47 (0.27–0.84)	0.48 (0.27–0.88)	46/49	1.04 (0.61–1.77)	1.18 (0.65–2.16)
> 7760	61/67	0.56 (0.32–0.98)	0.64 (0.35–1.17)	51/43	1.32 (0.75–2.32)	1.54 (0.82–2.89)
*P trend*		0.03	0.06		0.60	0.40
Per 1-SD increase		0.81 (0.66–1.01)	0.85 (0.67–1.09)		0.99 (0.79–1.24)	1.05 (0.81–1.36)
Leptin (pg/mL)						
< 1924	45/57	Reference	Reference	48/50	Reference	Reference
1924–4051	71/50	1.85 (1.06–3.23)	1.54 (0.82–2.87)	58/58	1.04 (0.61–1.79)	1.00 (0.55–1.84)
> 4051	42/51	1.08 (0.60–1.94)	0.60 (0.27–1.33)	60/58	1.08 (0.64–1.82)	0.99 (0.49–2.05)
*P trend*		0.06	0.01		0.97	1.00
Per 1-SD increase		1.07 (0.86–1.34)	0.86 (0.62–1.19)		0.99 (0.80–1.24)	0.90 (0.64–1.26)
Ghrelin (pg/mL)						
< 224	28/21	Reference	Reference	70/57	Reference	Reference
224–451	47/35	0.67 (0.33–1.39)	0.67 (0.29–1.51)	54/46	0.73 (0.40–1.36)	0.51 (0.25–1.07)
> 451	70/15	0.88 (0.29–2.64)	0.54 (0.13–2.29)	45/66	0.24 (0.10–0.56)	0.14 (0.05–0.41)
*P trend*		0.55	0.53		0.003	0.002
Per 1-SD increase		0.89 (0.60–1.34)	0.75 (0.46–1.25)		0.43 (0.29–0.63)	0.27 (0.16–0.47)
Androstenedione (pmol/L)						
< 2906	30/36	Reference	Reference	61/60	Reference	Reference
2906–4460	56/42	1.63 (0.81–3.28)	1.54 (0.70–3.38)	52/53	0.96 (0.53–1.73)	1.03 (0.53–2.00)
> 4460	59/67	1.10 (0.52–2.31)	1.06 (0.47–2.41)	30/30	0.97 (0.46–2.03)	0.88 (0.38–2.00)
*P trend*		0.23	0.35		0.99	0.90
Per 1-SD increase		1.09 (0.81–1.46)	1.12 (0.80–1.56)		0.98 (0.73–1.32)	0.99 (0.72–1.37)
DHEA (pmol/L)						
6593	46/38	Reference	Reference	55/53	Reference	Reference
6593–10,997	55/44	0.90 (0.48–1.70)	0.76 (0.37–1.55)	52/49	1.03 (0.58–1.85)	0.82 (0.43–1.58)
> 10,997	43/62	0.50 (0.25–0.98)	0.45 (0.21–0.95)	27/32	0.80 (0.40–1.60)	0.56 (0.25–1.30)
*P trend*		0.06	0.07		0.75	0.41
Per 1-SD increase		0.85 (0.65–1.11)	0.84 (0.62–1.13)		0.90 (0.67–1.20)	0.82 (0.58–1.15)
Estrone (pmol/L)						
< 110	41/45	Reference	Reference	53/51	Reference	Reference
110–150	49/52	1.07 (0.59–1.93)	1.00 (0.54–1.85)	48/44	1.04 (0.58–1.88)	0.94 (0.48–1.86)
> 150	55/48	1.30 (0.70–2.42)	1.12 (0.58–2.16)	42/48	0.82 (0.45–1.51)	0.82 (0.42–1.62)
*P trend*		0.66	0.91		0.72	0.85
Per 1-SD increase		1.03 (0.80–1.33)	0.96 (0.73–1.25)		0.93 (0.73–1.18)	0.90 (0.69–1.17)
Estradiol (pmol/L)						
< 61.4	37/36	Reference	Reference	60/60	Reference	Reference
61.4–82.7	47/52	0.88 (0.47–1.64)	0.83 (0.43–1.61)	47/44	1.08 (0.60–1.96)	1.08 (0.56–2.07)
> 82.7	61/57	1.03 (0.57–1.87)	0.89 (0.48–1.67)	36/39	0.93 (0.52–1.65)	0.92 (0.48–1.74)
*P trend*		0.82	0.86		0.89	0.90
Per 1-SD increase		1.01 (0.80–1.29)	0.95 (0.73–1.22)		1.07 (0.86–1.33)	1.02 (0.80–1.31)
Free estradiol (pmol/L)						
< 1.4	39/40	Reference	Reference	45/56	Reference	Reference
1.4–1.9	61/52	1.20 (0.65–2.22)	1.08 (0.56–2.09)	51/43	1.50 (0.83–2.70)	1.31 (0.69–2.46)
> 1.9	45/53	0.91 (0.49–1.66)	0.69 (0.35–1.36)	47/44	1.30 (0.74–2.30)	1.22 (0.64–2.33)
*P trend*		0.54	0.27		0.37	0.69
Per 1-SD increase		1.02 (0.81–1.28)	0.90 (0.69–1.17)		1.09 (0.88–1.35)	1.06 (0.83–1.34)
SHBG (nmol/L)^c^						
< 37.6	36/34	Reference	Reference	69/76	Reference	Reference
37.6–59.5	55/61	0.86 (0.46–1.60)	0.86 (0.43–1.70)	62/48	1.42 (0.84–2.42)	1.51 (0.83–2.74)
> 59.5	70/66	1.02 (0.51–2.02)	1.22 (0.56–2.68)	38/45	0.96 (0.52–1.77)	0.98 (0.48–1.98)
*P trend*		0.78	0.50		0.28	0.25
Per 1-SD increase		1.02 (0.78–1.33)	1.10 (0.81–1.50)		0.98 (0.75–1.28)	0.97 (0.72–1.31)
Testosterone (pmol/L)						
< 12,637	34/33	Reference	Reference	50/60	Reference	Reference
12,637–18,218	42/46	0.90 (0.49–1.67)	0.97 (0.48–1.97)	54/48	1.31 (0.78–2.19)	1.24 (0.70–2.21)
> 18,218	68/65	1.04 (0.54–2.01)	1.19 (0.55–2.56)	32/28	1.36 (0.72–2.58)	1.28 (0.62–2.63)
*P trend*		0.88	0.81		0.49	0.08
Per 1-SD increase		0.99 (0.78–1.26)	1.05 (0.80–1.37)		1.17 (0.88–1.56)	1.17 (0.85–1.62)
Free testosterone (pmol/L)						
< 209	51/46	Reference	Reference	41/47	Reference	Reference
209–267	44/41	0.96 (0.54–1.69)	0.97 (0.51–1.83)	55/52	1.25(0.69–2.25)	1.27 (0.65–2.49)
> 267	49/57	0.74 (0.41–1.34)	0.76 (0.39–1.46)	40/37	1.32 (0.69–2.54)	1.62 (0.76–3.43)
*P trend*		0.57	0.65		0.66	0.45
Per 1-SD increase		0.99 (0.79–1.25)	0.99 (0.77–1.27)		1.18 (0.90–1.54)	1.20 (0.90–1.59)
Progesterone (pmol/L)						
< 130		NM	NM	47/47	Reference	Reference
130–202				54/47	1.12 (0.64–1.97)	1.21 (0.64–2.27)
> 202				42/49	0.85 (0.46–1.57)	0.97 (0.48–1.95)
*P trend*					0.62	0.73
Per 1-SD increase					0.98 (0.77–1.25)	1.01 (0.76–1.33)

All variables were log transformed

Not all of the cases/controls will sum to the total due to missing data

*CGC* cardia gastric cancer, *CI* confidence interval, *DHEA* dehydroepiandrosterone, *IGF-1* insulin-like growth factor-1, *IGFBP-3* insulin-like growth factor-binding protein-3, *NCGC* non-cardia gastric cancer, *NM* not measured, *OR* odds ratio, *SD* standard deviation, *SHBG* sex hormone-binding globulin

^a^Crude model was integrally adjusted for the matching factors, including study center, sex, age at recruitment and date/time of blood collection in EPIC and age at randomization and date of blood draw in ATBC

^b^Adjusted model was based on crude model with further adjustment for education level, study center, smoking and body mass index

^c^Additionally adjusted for insulin

In the UK-Biobank, SHBG was positively associated with CGC in men (adjusted HR 1.90, 95% CI 1.04–3.47 for the highest vs. lowest category, and HR 1.29, 95% CI 1.02–1.64 per 1-SD increase) (Table 5). For NCGC, an inverse association was observed with free testosterone in men in continuous data only (per 1-SD increase: adjusted HR 0.71, 95% CI 0.57–0.89). In UK-Biobank women, similar to EPIC men, a positive association was observed between IGF-1 and CGC (adjusted HR 5.13, 95% CI 1.26–20.88 for the highest vs. lowest category and HR 1.76, 95% CI 1.08–2.88 per 1-SD increase) (Table 6). For hormones without sex differences, no significant associations were observed in sex-combined analyses (Supplementary table 6).

**Table 5:** Hazard ratios and 95% confidence intervals for circulating hormones and gastric cancer by subsite in men from the UK-Biobank study

	CGC			NCGC		
	Cases (*N* = 113)	Crude HR^a^ (95% CI)	Adjusted HR^b^ (95% CI)	Cases (*N* = 57)	Crude HR^a^ (95% CI)	Adjusted HR^b^ (95% CI)
Glucose (mmol/L)						
< 4.7	30	Reference	Reference	15	Reference	Reference
4.7–5.2	29	0.94 (0.55–1.62)	0.97 (0.56–1.67)	16	1.12 (0.54–2.33)	1.13 (0.54–2.36)
> 5.2	39	1.09 (0.65–1.82)	1.07 (0.63–1.80)	17	1.03 (0.50–2.13)	1.07 (0.51–2.24)
*P trend*		0.85	0.93		0.95	0.95
Per 1-SD increase		1.17 (0.99–1.38)	1.13 (0.95–1.34)		1.03 (0.79–1.35)	1.06 (0.80–1.39)
HbA1c (mmol/mol)						
< 33.6	28	Reference	Reference	16	Reference	Reference
33.6–37.0	26	0.86 (0.49–1.51)	0.77 (0.43–1.37)	13	0.83 (0.39–1.77)	0.86 (0.40–1.85)
> 37.0	49	1.27 (0.76–2.11)	0.99 (0.59–1.68)	25	1.19 (0.60–2.33)	1.24 (0.62–2.49)
*P trend*		0.29	0.57		0.58	0.57
Per 1-SD increase		1.14 (0.94–1.37)	1.04 (0.85–1.27)		1.16 (0.93–1.46)	1.19 (0.95–1.50)
CRP (mg/L)						
< 0.8	22	Reference	Reference	13	Reference	Reference
0.8–1.9	40	1.42 (0.82–2.45)	1.15 (0.66–2.00)	16	1.17 (0.55–2.50)	1.22 (0.57–2.63)
> 1.9	44	1.60 (0.94–2.72)	1.10 (0.63–1.94)	24	1.48 (0.73–3.03)	1.58 (0.74–3.36)
*P trend*		0.23	0.89		0.53	0.47
Per 1-SD increase		1.25 (1.03–1.53)	1.10 (0.88–1.37)		1.21 (0.92–1.59)	1.23 (0.93–1.64)
IGF-1 (nmol/L)						
< 19.6	44	Reference	Reference	27	Reference	Reference
19.6–23.9	31	0.87 (0.53–1.43)	0.93 (0.57–1.52)	15	0.70 (0.37–1.33)	0.69 (0.36–1.32)
> 23.9	31	0.99 (0.60–1.62)	1.06 (0.64–1.75)	11	0.50 (0.23–1.07)	0.50 (0.23–1.08)
*P trend*		0.85	0.89		0.18	0.17
Per 1-SD increase		1.00 (0.82–1.22)	1.04 (0.85–1.27)		0.77 (0.60–1.00)	0.77 (0.59–1.00)
SHBG (nmol/L)						
< 30.9	19	Reference	Reference	9	Reference	Reference
30.9–43.7	34	1.42 (0.79–2.55)	1.68 (0.92–3.06)	13	1.13 (0.47–2.71)	1.11 (0.46–2.66)
> 43.7	46	1.43 (0.81–2.55)	1.90 (1.04–3.47)	26	1.95 (0.89–4.26)	1.89 (0.84–4.27)
*P trend*		0.41	0.11		0.14	0.18
Per 1-SD increase		1.16 (0.92–1.45)	1.29 (1.02–1.64)		1.38 (1.01–1.89)	1.36 (0.98–1.90)
Testosterone (nmol/L)						
< 10.2	38	Reference	Reference	16	Reference	Reference
10.2–13.2	32	0.83 (0.50–1.37)	0.99 (0.59–1.65)	11	0.59 (0.26–1.33)	0.61 (0.27–1.39)
> 13.2	37	1.00 (0.62–1.62)	1.28 (0.77–2.13)	25	1.55 (0.82–2.92)	1.58 (0.81–3.11)
*P trend*		0.70	0.55		0.04	0.05
Per 1-SD increase		0.97 (0.79–1.19)	1.08 (0.87–1.35)		0.94 (0.72–1.23)	0.91 (0.69–1.22)
Free testosterone (pmol/L)						
< 181	48	Reference	Reference	19	Reference	Reference
181–227	27	0.68 (0.42–1.12)	0.73 (0.44–1.20)	20	1.39 (0.71–2.70)	1.37 (0.70–2.68)
> 227	23	0.73 (0.42–1.27)	0.82 (0.47–1.45)	8	0.72 (0.31–1.71)	0.73 (0.30–1.73)
*P trend*		0.26	0.45		0.28	0.30
Per 1-SD increase		0.85 (0.70–1.03)	0.90 (0.73–1.10)		0.73 (0.58–0.91)	0.71 (0.57–0.89)

^a^Crude model was stratified on age, center and town send deprivation index

^b^Adjusted model was based on crude model with further adjustment for education level, smoking, and body mass index

Not all of the cases will sum to the total due to missing data

*CGC* cardia gastric cancer, *CI* confidence interval, *CRP* C-reactive protein, *HbA1c* glycated hemoglobin, *HR* hazard ratio, *IGF-1* insulin-like growth factor-1, *NCGC* non-cardia gastric cancer, *SD* standard deviation, *SHBG* sex hormone-binding globulin

**Table 6:** Hazard ratios and 95% confidence intervals for circulating hormones and gastric cancer by subsite in women from the UK-Biobank study

	CGC			NCGC		
	Cases (*N* = 24)	Crude HR^a^ (95% CI)	Adjusted HR^b^ (95% CI)	Cases (*N* = 35)	Crude HR^a^ (95% CI)	Adjusted HR^b^ (95% CI)
Glucose (mmol/L)						
< 4.7	7	Reference	Reference	6	Reference	Reference
4.7–5.1	6	1.19 (0.37–3.83)	1.19 (0.37–3.86)	14	2.06 (0.77–5.52)	2.10 (0.78–5.65)
> 5.1	6	0.97 (0.28–3.39)	1.03 (0.29–3.64)	13	1.91 (0.72–5.12)	1.89 (0.70–5.10)
*P trend*		0.94	0.95		0.33	0.32
Per 1-SD increase		1.02 (0.63–1.66)	1.02 (0.63–1.66)		1.21 (0.97–1.52)	1.19 (0.95–1.51)
HbA1c (mmol/mol)						
< 33.5	7	Reference	Reference	8	Reference	Reference
33.5–36.7	11	1.15 (0.40–3.24)	1.12 (0.39–3.20)	12	0.93 (0.37–2.33)	0.91 (0.36–2.28)
> 36.7	6	0.59 (0.18–1.90)	0.54 (0.16–1.78)	15	0.96 (0.39–2.36)	0.87 (0.35–2.16)
*P trend*		0.44	0.37		0.99	0.95
Per 1-SD increase		0.81 (0.50–1.32)	0.79 (0.48–1.30)		1.13 (0.86–1.49)	1.11 (0.83–1.50)
CRP (mg/L)						
< 0.8	9	Reference	Reference	7	Reference	Reference
0.8–2.2	7	0.75 (0.26–2.17)	0.74 (0.25–2.19)	15	1.61 (0.65–4.01)	1.53 (0.60–3.90)
> 2.2	8	0.77 (0.27–2.17)	0.69 (0.21–2.21)	13	1.30 (0.51–3.34)	1.24 (0.44–3.48)
*P trend*		0.84	0.80		0.59	0.65
Per 1-SD increase		0.93 (0.59–1.45)	0.88 (0.53–1.46)		1.01 (0.71–1.44)	0.97 (0.65–1.46)
IGF-1 (nmol/L)						
< 18.3	3	Reference	Reference	12	Reference	Reference
18.3–23.2	11	4.61 (1.26–16.96)	4.98 (1.33–18.65)	13	1.38 (0.61–3.15)	1.37 (0.60–3.13)
> 23.2	9	4.68 (1.19–18.46)	5.13 (1.26–20.88)	10	1.61 (0.67–3.88)	1.64 (0.68–3.95)
*P trend*		0.05	0.04		0.54	0.54
Per 1-SD increase		1.73 (1.07–2.79)	1.76 (1.08–2.88)		1.11 (0.78–1.57)	1.11 (0.78–1.58)
SHBG (nmol/L)						
< 45.4	8	Reference	Reference	13	Reference	Reference
45.4–69.0	3	0.41 (0.11–1.53)	0.40 (0.10–1.57)	10	0.86 (0.37–1.99)	0.85 (0.35–2.03)
> 69.0	8	0.86 (0.29–2.51)	0.82 (0.25–2.76)	10	0.80 (0.34–1.92)	0.79 (0.30–2.06)
*P trend*		0.41	0.42		0.88	0.88
Per 1-SD increase		0.75 (0.46–1.21)	0.69 (0.40–1.17)		0.89 (0.63–1.26)	0.88 (0.60–1.29)
Testosterone (nmol/L)						
< 0.8	10	Reference	Reference	9	Reference	Reference
0.8–1.2	6	0.66 (0.23–1.90)	0.66 (0.23–1.92)	9	1.43 (0.53–3.86)	1.45 (0.54–3.90)
> 1.2	5	0.55 (0.18–1.68)	0.52 (0.17–1.63)	7	1.23 (0.43–3.53)	1.25 (0.43–3.60)
*P trend*		0.53	0.50		0.78	0.77
Per 1-SD increase		0.75 (0.47–1.18)	0.74 (0.47–1.17)		1.26 (0.85–1.89)	1.27 (0.85–1.90)
Free testosterone (pmol/L)						
< 9.6	9	Reference	Reference	4	Reference	Reference
9.6–15.8	5	0.56 (0.18–1.76)	0.60 (0.19–1.93)	13	4.33 (1.22–15.38)	4.58 (1.28–16.44)
> 15.8	4	0.44 (0.13–1.51)	0.42 (0.11–1.56)	7	2.57 (0.66–10.00)	2.95 (0.73–11.97)
*P trend*		0.37	0.40		0.07	0.06
Per 1-SD increase		0.81 (0.50–1.32)	0.80 (0.48–1.34)		1.37 (0.90–2.08)	1.47 (0.95–2.28)

Not all of the cases will sum to the total due to missing data

*CGC* cardia gastric cancer, *CI* confidence interval, *CRP* C-reactive protein, *HbA1c* glycated hemoglobin, *HR* hazard ratio, *IGF-1* insulin-like growth factor-1, *NCGC* non-cardia gastric cancer, *SD* standard deviation, *HBG* sex hormone-binding globulin

^a^Crude model was stratified on age, center and town send deprivation index

^b^Adjusted model was based on crude model with further adjustment for education level, smoking, and body mass index

In EPIC, we found strong to moderate correlations between androstenedione and DHEA (0.71), estrone and estradiol (0.76), testosterone and estradiol (0.79) and, as expected, between testosterone and free testosterone (0.99), and estradiol and free estradiol (0.99) (Supplementary table 7). Similar correlations were observed between sex hormones in ATBC (Supplementary table 8). In UK-Biobank, the only strong correlation was between testosterone and free testosterone (0.79) (Supplementary table 9). In models mutually adjusted for (1) all of the gut/adipokines and (2) all of the sex hormones, the direction and magnitude of the associations described remained the same, with the exception of IGF-1 and CGC, and of leptin and NCGC in EPIC men, and C-peptide and NCGC in EPIC women, which attenuated and were no longer statistically significant.

Sensitivity analysis excluding CGC and NCGC cases diagnosed during the first year of follow-up did not substantially change the results (data not shown).

## Discussion

In our analyses, IGF-1 was positively associated with CGC and NCGC in men in EPIC; similar findings were observed for IGF-1 and CGC in women in UK-Biobank, although there was no association in ATBC. In EPIC, leptin was positively associated with NCGC in men, while C-peptide was positively associated with NCGC in women. For ghrelin, we observed inverse associations for NCGC among men in EPIC and ATBC. In addition, insulin was inversely associated with NCGC in men in ATBC. With regard to sex hormones, DHEA was inversely associated with CGC in EPIC and ATBC men combined, and free testosterone was inversely associated with NCGC in men in UK-Biobank only. In contrast, SHBG was positively associated with CGC in UK-Biobank men only.

Results from previous nested case–control studies and a cohort study reported no association between IGF-1 and gastric cancer risk [12–14], although these studies did not examine the associations for CGC and NCGC separately. In our study, findings for IGF-1 were intriguing but inconsistent, with positive associations observed for both CGC and NCGC subsites in EPIC men but only for CGC among women in UK-Biobank, and no associations in ATBC. Experimental studies suggest IGF-1 may play a role in the development of gastric cancer [34, 35]. IGF-1 is thought to promote cancer development by stimulating cell proliferation and inhibiting apoptosis [34, 35].

Ghrelin was inversely associated with NCGC in men in EPIC and ATBC. Two previous nested case–control studies observed this same effect [19, 20]. Our results for ghrelin and NCGC in ATBC replicate findings of a previous ATBC study, which investigated the association between serum ghrelin concentration and risk of gastric non-cardia adenocarcinoma (*n* = 261 cases) and esophagogastric junctional adenocarcinoma (*n* = 98 cases) [19]. The potential mechanisms underlying an association between ghrelin and gastric cancer are unclear; however, a recent study found that ghrelin inhibits cell proliferation, migration, and invasion in gastric cancer cells [36]. Conversely, in another study, ghrelin induced cell proliferation, migration and invasion in gastric cancer cells [37]. For leptin, a satiety hormone, we observed a positive association with NCGC in EPIC men only. Case–control studies have reported lower serum leptin levels in gastric cancer patients compared to controls [18, 38] but these studies did not provide data on the association between leptin levels and gastric cancer risk and did not examine leptin and gastric cancer subsite. Mechanistically, leptin can stimulate cell proliferation in gastric cancer cells, which may contribute to cancer development [39].

Insulin was inversely associated with NCGC only in ATBC, whereas C-peptide was positively associated with NCGC in EPIC women. A prospective case–control study reported a positive association between insulin and early gastric cancer risk [40] but did not analyze by cancer subtype; although a nested case–control study showed a positive association between both C-peptide and insulin for NCGC but the risk estimates were not significant [10]. Regarding CGC, a large prospective cohort study (NIH-AARP Diet and Health study) has previously shown a positive association between self-reported diabetes and CGC [41] but there is little data on insulin and CGC [42]. The role of insulin in other malignancies was established with a meta-analysis reporting an increased risk for colorectal and pancreatic cancers with higher levels of insulin and C-peptide [43]. Despite the lack of epidemiological data, a recent in vitro study demonstrated that insulin exhibits direct cancer-promoting effects on gastric cancer cells [44].

For the sex hormones in our study, DHEA was inversely associated with CGC in men from EPIC and ATBC combined. A previous nested case–control study in men showed DHEA was associated with a 38% decreased risk of esophageal/gastric adenocarcinoma combined [24]; whilst a case–control study reported an inverse association between DHEA and NCGC in sex-combined analysis [45]. DHEA has been shown to have a protective role against cancer [46, 47]. Experimental studies have demonstrated that DHEA inhibits proliferation of cancer cells [46] and derivatives of DHEA exhibit anti-tumor activity against gastric cancer cells [47].

We also observed an inverse association between free testosterone and NCGC among men in UK-Biobank only, although a previous nested case–control study showed no association between testosterone and NCGC [25]. For SHBG, we observed a positive association with CGC among men in UK-Biobank only. A study nested within three US cohort studies showed no association between SHBG and esophageal and gastric adenocarcinoma combined [24], whilst a previous UK-Biobank study and a nested case–control study showed a positive association between SHBG concentrations and NCGC [25, 26]. Few studies have examined the association between free testosterone or SHBG with risk of CGC and NCGC. SHBG is a glycoprotein, which binds circulating androgens and estrogens and regulates the bioavailability of androgens and estrogens [48]. Estrogen can decrease cell viability and induce apoptosis in gastric cancer cells [49]. Additionally, polymorphisms in SHBG and catechol-O-methyltransferase, involved in estrogen inactivation, have been associated with gastric cancer risk [50].

Evidence from both experimental and observational studies suggest plausible mechanisms to explain observed associations between circulating hormones and gastric cancer risk. However, consistent with the fact that risk factor profiles differ by cancer subsite, some of the hormone associations also appear to vary by gastric cancer subsite.

Our study had many strengths, including its prospective design with serum samples and questionnaire data collected before cancer diagnosis, data available on potential confounders, and the opportunity to measure several hormones. Nevertheless, there are limitations in our study. Residual confounding may affect our data; in particular, we had no information on *Helicobacter pylori* infection, which is a well-known risk factor for NCGC. The enrollment years vary across the three studies, which could have influenced risk factor exposure: the changing prevalence of *Helicobacter pylori* infection over time [51], and the rise in obesity and its implication for CGC cancer [3]. In the ATBC study, participants were male smokers, which may limit the generalizability of findings from that study. Most importantly, despite using three large cohorts with long follow-up periods, the number of cases and controls in some analyses remained small; hence, our results should be interpreted with caution. Although we examined several variables, we did not adjust for multiple comparisons given that the analytes were selected *a-priori,* and each had specific proposed mechanisms through which they may be associated with gastric cancer. Finally, hormone levels were measured only at one time-point (baseline), which could lead to misclassification if hormone levels changed over the course of follow-up.

In conclusion, we found IGF-1 and SHBG were positively associated with CGC, while IGF-1, C-peptide and leptin were positively associated with NCGC. Furthermore, DHEA was inversely associated with CGC, whereas ghrelin and free testosterone were inversely associated with NCGC. Our findings suggest that some obesity-related hormones may influence CGC and NCGC risk. Further prospective studies with larger sample size are needed to examine endogenous hormones measurements in relation to gastric cancer risk by subsite.

### Supplementary Information

Below is the link to the electronic supplementary material.

Supplementary file1 (DOCX 82 KB)

## Data Availability

For information on how to submit an application for gaining access to EPIC data and/or biospecimens, please follow the instructions at http://epic.iarc.fr/access/index.php. The UK Biobank is an open access resource and bona fide researchers can apply to use the UK Biobank data set by registering and applying at http://ukbiobank.ac.uk/register-apply/. Further information is available from the corresponding author upon request.

## Abbreviations

ATBC: Alpha-tocopherol, beta-carotene cancer prevention
BMI: Body mass index
CGC: Cardia gastric cancer
CRP: C-reactive protein
CI: Confidence intervals
DHEA: Dehydroepiandrosterone
EPIC: European Prospective Investigation into Cancer and Nutrition
HR: Hazard ratio
HbA1c: Glycated hemoglobin
IGF-1: Insulin-like growth factor 1
IGFBP-3: Insulin-like growth factor-binding protein 3
NCGC: Non-cardia gastric cancer
OR: Odds ratio
SD: Standard deviation
SHBG: Sex hormone-binding globulin
