# Supplementary material for: Circulating hormones and risk of gastric cancer by subsite in three cohort studies
Source: Gastric Cancer. 2023 Jul 16;26(6):969–87. doi: 10.1007/s10120-023-01414-0 (PMC10640529; doi:10.1007/s10120-023-01414-0)
Supplement: Supplementary file 1 — Supplementary file1 (DOCX 82 KB) [file 10120_2023_1414_MOESM1_ESM.docx]

Supplementary table 1. Selected characteristics of participating cohorts

| **Study** | **Location** | **Enrolment years** | **Baseline cohort** | **Age at enrolment**  **years** | **Follow-up (mean)**  **years** | **Study design** | **Cases/**  **Controls** | **Hormone measurements** | **Matching** |
| --- | --- | --- | --- | --- | --- | --- | --- | --- | --- |
| **European Prospective Investigation into Cancer and Nutrition (EPIC) Cohort** | Europe | 1992-2000 | 521,324 men/women | 25-70 | 14 | Nested case-control study | CGC-61/61  NCGC-172/172 | Insulin, C-peptide, IGF-1, IGFBP-3, adiponectin, leptin, ghrelin, androstenedione,  DHEA, estrone, estradiol, SHBG, testosterone, and progesterone | Study recruitment center, age at recruitment, gender, and date and time of the day at blood collection |
| **Alpha-Tocopherol, Beta-Carotene Cancer Prevention (ATBC) Study** | Finland | 1985-1988 | 29,133 men | 50-69 | 20 | Nested case-control study | CGC-100/100  NCGC-65/65 | Insulin, IGF-1, IGFBP-3, adiponectin, leptin, ghrelin, androstenedione, androsterone  DHEA, estrone, estradiol, SHBG, dihydrotestosterone testosterone and progesterone | Age at randomization, and date of blood draw |
| **UK-Biobank cohort** | UK | 2006-2010 | 502,524 men/women | 40-69 | 6.5 | Cohort | CGC-137  NCGC-92 | Glucose, HbA1c, CRP, IGF-1, SHBG, and testosterone | N/A |

Abbreviations: CGC, cardia gastric cancer; CRP, C-reactive protein; DHEA, dehydroepiandrosterone; HbA1c, glycated hemoglobin; IGF-1, insulin-like growth factor-1; IGFBP-3, insulin-like growth factor-binding protein-3; NCGC, non-cardia gastric cancer; SHBG, sex hormone-binding globulin

Supplementary table 2. Baseline characteristics of cases and controls in the EPIC study

| **EPIC (men and women)** | **CGC** | | |  |  | **NCGC** | | | | |
| --- | --- | --- | --- | --- | --- | --- | --- | --- | --- | --- |
|  | **Cases** | | **Controls** | **P**  **value** |  | **Cases** | | **Controls** | | **P**  **value** |
| **Total (N)** | | 61 | 61 |  |  | 172 | 172 | |  | |
| **Men (N)** | | 61 | 61 |  |  | 104 | 104 | |  | |
| **Age at recruitment* (years)** | | 58.2±8.3 | 58.7±8.1 | 0.75 |  | 57.3±7.1 | 57.2±7.1 | | 0.88 | |
| **Time between blood collection and cancer diagnosis† (years)** | | 8.0 | - |  |  | 8.4 | - | |  | |
| **BMI* (kg/m^2^)** | | 27.1±3.3 | 27.2±4.4 | 0.85 |  | 27.8±3.8 | 27.4±4.0 | | 0.32 | |
| **Education level (%)** |  | |  | 0.54 |  |  |  | | 0.006 | |
| None | 1.6 | | 1.6 |  |  | 12.8 | 17.4 | |  | |
| Primary school | 36.1 | | 26.2 |  |  | 53.4 | 33.7 | |  | |
| Technical/professional | 31.2 | | 31.2 |  |  | 15.7 | 19.8 | |  | |
| Secondary school | 4.9 | | 13.11 |  |  | 6.4 | 13.9 | |  | |
| University degree | 16.4 | | 21.31 |  |  | 9.9 | 13.9 | |  | |
| Missing/not specified | 9.8 | | 6.6 |  |  | 1.7 | 1.2 | |  | |
| **Smoking (%)** |  | |  | 0.79 |  |  |  | | 0.63 | |
| Never | 22.9 | | 27.9 |  |  | 43.0 | 47.7 | |  | |
| Former | 37.7 | | 42.6 |  |  | 27.9 | 29.1 | |  | |
| Current | 36.1 | | 27.9 |  |  | 27.9 | 20.9 | |  | |
| Unknown/missing | 3.3 | | 1.6 |  |  | 1.2 | 2.3 | |  | |
| **Dietary variables*** |  | |  |  |  |  |  | |  | |
| Alcohol (g/day) | 16.7±18.8 | | 18.2±20.9 | 0.68 |  | 18.9±23.5 | 19.4±22.2 | | 0.84 | |
| Vegetable (g/day) | 174±105 | | 200±127 | 0.22 |  | 188±131 | 181±122 | | 0.62 | |
| Fruit (g/day) | 208±216 | | 225±153 | 0.62 |  | 292±209 | 267±240 | | 0.31 | |
| Red meat (g/day) | 54.4±39.5 | | 41.7±28.3 | 0.04 |  | 47.4±32.5 | 49.4±34.5 | | 0.58 | |
| Processed meat (g/day) | 42.2±32.9 | | 42.5±45.5 | 0.96 |  | 41.6±43.2 | 42.9±39.2 | | 0.77 | |
| Energy (kcal/day) | 2249±715 | | 2233±587 | 0.89 |  | 2252±584 | 2278±632 | | 0.69 | |
| **Serologic variables*** |  | |  |  |  |  |  | |  | |
| Insulin (pg/mL) | | 371±232 | 352±168 | 0.79 |  | 360±350 | 299±159 | | 0.16 | |
| C-peptide (pmol/L) | | 930±601 | 862±457 | 0.48 |  | 893±532 | 825±572 | | 0.26 | |
| IGF-1 (ng/mL) | | 103±31.4 | 95.5±28.9 | 0.15 |  | 88.5±29.4 | 88±31 | | 0.80 | |
| IGFBP-3 (ng/mL) | 2028±520 | | 2058±501 | 0.75 |  | 2118±459 | 2240±469 | | 0.02 | |
| Adiponectin (ng/mL) | 6903±3030 | | 7083±3402 | 0.76 |  | 8101±4535 | 8639±5421 | | 0.32 | |
| Leptin (pg/mL) | 4255±3549 | | 5152±6409 | 0.35 |  | 9781±11937 | 8699±10666 | | 0.38 | |
| Ghrelin (pg/mL) | 258±160 | | 274±141 | 0.57 |  | 236±141 | 283±189 | | 0.009 | |
| Androstenedione (pmol/L) | | 3087±1438 | 2841±1165 | 0.37 |  | 2459±1152 | 2612±1353 | | 0.33 | |
| DHEA (pmol/L) | 7340±4141 | | 7674±5857 | 0.76 |  | 7484±4338 | 7934±4965 | | 0.44 | |
| Estrone (pmol/L) | 131±52.3 | | 124±40.8 | 0.54 |  | 109±78.1 | 110±99.1 | | 0.95 | |
| Estradiol (pmol/L) | 69.2±24.3 | | 67.2±19.1 | 0.65 |  | 56.3±87.9 | 56.3±134 | | 0.99 | |
| Free estradiol (pmol/L) | 1.7±0.52 | | 1.7±0.52 | 0.80 |  | 1.4±2.0 | 1.3±2.3 | | 0.73 | |
| SHBG (nmol/L) | 44.7±18.7 | | 41.5±16.6 | 0.32 |  | 45.8±20.7 | 47.8±23.0 | | 0.40 | |
| Testosterone (pmol/L) | 14287±4776 | | 13222±3637 | 0.24 |  | 8515±7271 | 8474±7478 | | 0.96 | |
| Free testosterone (pmol/L) | | 229±63.1 | 226±61.9 | 0.82 |  | 150±126 | 146±123 | | 0.78 | |
| Progesterone (pmol/L) | 218±246 | | 193±156 | 0.56 |  | 2623±1309 | 159±155 | | 0.37 | |

*Mean ± SD. †Median. Abbreviations: BMI, body mass index; CGC, cardia gastric cancer; DHEA, dehydroepiandrosterone; EPIC, European Prospective Investigation into Cancer and Nutrition; IGF-1, insulin-like growth factor-1; IGFBP-3, insulin-like growth factor-binding protein-3; NCGC, non-cardia gastric cancer; SD, standard deviation; SHBG, sex hormone-binding globulin

Supplementary table 3. Baseline characteristics of cases and controls in the ATBC study

| **ATBC (men)** | | **CGC** | | |  | **NCGC** | | |  |
| --- | --- | --- | --- | --- | --- | --- | --- | --- | --- |
|  | | **Cases** | **Controls** | **P value** |  | **Cases** | **Controls** | **P**  **value** |  |
| **Total Men (N)** | | 100 | 100 |  |  | 65 | 65 |  |  |
| **Age at randomization*(years)** | | 58.2±4.8 | 58.2±4.8 | 0.99 |  | 58.3±5.2 | 58.2±4.9 | 0.93 |  |
| **Time between blood collection and cancer diagnosis† (years)** | | 9.0 | - |  |  | 7.0 | - |  |  |
| **BMI* (kg/m^2^)** | | 26.8±3.9 | 25.6±3.4 | 0.02 |  | 26.3±3.7 | 26.9±4.3 | 0.38 |  |
| **Education: (%)** | |  |  |  |  |  |  |  |  |
| 8^th^ grade or less | | 28.0 | 29.0 | 1.0 |  | 29.2 | 30.8 | 1.0 |  |
| Less than high school | | - | - |  |  | 1.5 | - |  |  |
| College or technical school | | 67.0 | 33.0 |  |  | 63.1 | 61.5 |  |  |
| College graduate | | 5.0 | 2.5 |  |  | 6.2 | 7.7 |  |  |
| **Smoking*** | |  |  |  |  |  |  |  |  |
| Years of smoking | | 37.1±6.8 | 36.9±6.8 | 0.88 |  | 38.4±8.1 | 37.3±7.6 | 0.44 |  |
| Cigarettes/day | | 21.3±9.1 | 20.9±9.3 | 0.77 |  | 21.4±9.3 | 20.1±8.3 | 0.42 |  |
| **Dietary variables*** | |  |  |  |  |  |  |  |  |
| Alcohol (g/day) | | 15.7±16.3 | 15.9±17.4 | 0.92 |  | 17.2±17.3 | 18.8±16.7 | 0.60 |  |
| Vegetable (g/day) | | 79.7±54.2 | 88.6±61.2 | 0.29 |  | 76.5±49.7 | 96.9±53.7 | 0.03 |  |
| Fruit (g/day) | | 214±165 | 195±143 | 0.39 |  | 209±161 | 227±181 | 0.55 |  |
| Red meat (g/day) | | 70.0±36.2 | 73.2±36.5 | 0.56 |  | 71.8±27.2 | 71.2±28.4 | 0.91 |  |
| Processed meat (g/day) | | 74.8±46.9 | 68.7±55.3 | 0.42 |  | 71.4±51.3 | 59.9±50.9 | 0.22 |  |
| Energy (kcal/day) | | 2641±680 | 2578±696 | 0.53 |  | 2707±760 | 2567±639 | 0.28 |  |
| **Serologic variables*** | |  |  |  |  |  |  |  |  |
| Insulin (pg/mL) | | 318±321 | 357±729 | 0.62 |  | 277±151 | 356±224 | 0.02 |  |
| IGF-1 (ng/mL) | | 93.3±24.5 | 98.4±25.8 | 0.15 |  | 99.7±28.8 | 96.9±25.8 | 0.56 |  |
| IGFBP-3 (ng/mL) | | 17866±495 | 1796±529 | 0.88 |  | 1987±512 | 1977±495 | 0.91 |  |
| Adiponectin (ng/mL) | | 7389±3123 | 8476±3754 | 0.03 |  | 7214±2705 | 7071±2979 | 0.78 |  |
| Leptin (pg/mL) | | 3539±4048 | 3386±3963 | 0.79 |  | 3879±4932 | 5002±6000 | 0.25 |  |
| Ghrelin (pg/mL) | | 604±213 | 788±298 | 0.02 |  | 556±213 | 807±363 | <0.0001 |  |
| Androstenedione (pmol/L) | | 4818±1663 | 4836±1519 | 0.94 |  | 4013±1391 | 4091±1816 | 0.78 |  |
| Androsterone (pmol/L) | | 985±463 | 904±307 | 0.18 |  | - | - | - |  |
| DHEA (pmol/L) | | 10439±5236 | 11969±6025 | 0.06 |  | 8097±3939 | 9201±5150 | 0.19 |  |
| Estrone (pmol/L) | | 145±45 | 149±61 | 0.66 |  | 152±71 | 158±61 | 0.61 |  |
| Estradiol (pmol/L) | | 85.3±28.4 | 85.6±28.3 | 0.92 |  | 73.4±30.6 | 77.3±29.8 | 0.47 |  |
| Free estradiol (pmol/L) | | 1.8±0.6 | 1.8±0.6 | 0.91 |  | 1.7±0.8 | 1.7±0.6 | 0.80 |  |
| SHBG (nmol/L) | | 70.5±31.1 | 73.2±33 | 0.56 |  | 56.7±24.3 | 59.5±31.8 | 0.57 |  |
| Testosterone (pmol/L) | | 20979±8043 | 21917±8467 | 0.42 |  | 17012±5778 | 16445±6801 | 0.62 |  |
| Free testosterone (pmol/L) | | 259±80.4 | 265±75.8 | 0.56 |  | 242±95.3 | 233±84.3 | 0.59 |  |
| Dihydrotestosterone (pmol/L) | 1709±772 | | 1627±673 | 0.43 |  | - | - | - |  |
| Progesterone (pmol/L) | | - | - | - |  | 192±104 | 201±99.6 | 0.61 |  |

*Mean ± SD. †Median. Abbreviations: ATBC, Alpha-Tocopherol, Beta-Carotene Cancer Prevention; BMI, body mass index; CGC, cardia gastric cancer; DHEA, dehydroepiandrosterone; IGF-1, insulin-like growth factor-1; IGFBP-3, insulin-like growth factor-binding protein-3; NCGC, non-cardia gastric cancer; SD, standard deviation; SHBG, sex hormone-binding globulin

Supplementary table 4. Baseline characteristics of cases and non-cases in the UK-Biobank study

| **UK-Biobank (men and women)** | **CGC** | | |  | **NCGC** | | | |
| --- | --- | --- | --- | --- | --- | --- | --- | --- |
|  | **Cases** | **Non-cases** | **P value** |  | **Cases** | **Non-cases** | | **P value** |
| **Total (N)** | 137 | 458576 |  |  | 92 | 458621 | |  |
| **Men (N)** | 113 | 214180 |  |  | 57 | 214236 | |  |
| **Age at recruitment* (years)** | 61.8±6.3 | 56.2±8.1 | <.0001 |  | 60.7±6.9 | 56.2±8.1 | | <0.0001 |
| **Time between blood collection and cancer diagnosis† (years)** | 2.8 | - |  |  | 3.2 | - | |  |
| **BMI* (kg/m^2^)** | 28.7±4.9 | 27.4±4.8 | <.0001 |  | 27.7±5.0 | 27.4±4.8 | | 0.63 |
| **Education (%)** |  |  |  |  |  |  | |  |
| None | 30.7 | 16.6 | 0.0002 |  | 33.7 | 16.6 | | 0.001 |
| CSEs/O-levels/GCSEs or equivalent | 18.3 | 26.3 |  |  | 23.9 | 26.3 | |  |
| Vocational professional | 16.8 | 17.6 |  |  | 14.1 | 17.6 | |  |
| Other professional | 7.3 | 5.1 |  |  | 3.3 | 5.1 | |  |
| College/university | 23.4 | 32.4 |  |  | 21.7 | 32.4 | |  |
| Missing/unknown | 3.7 | 2.2 |  |  | 3.3 | 2.2 | |  |
| **Smoking status (%)** |  |  |  |  |  |  | |  |
| Never | 30.7 | 54.7 | <.0001 |  | 43.5 | 54.7 | | 0.16 |
| Former | 45.9 | 33.9 |  |  | 41.3 | 33.9 | |  |
| Current | 20.4 | 10.6 |  |  | 14.1 | 10.6 | |  |
| Missing/unknown | 2.9 | 0.7 |  |  | 1.1 | 0.7 | |  |
| **Alcohol intake frequency (%)** |  |  |  |  |  |  | |  |
| Never | 13.4 | 8.0 | <.0001 |  | 9.8 | 8.0 | | 0.02 |
| Special occasions only | 7.3 | 11.4 |  |  | 15.2 | 11.4 | |  |
| 1-3 times/month | 15.3 | 11.1 |  |  | 10.9 | 11.1 | |  |
| 1-2 times/week | 20.4 | 25.7 |  |  | 33.7 | 25.7 | |  |
| 3-4 times/week | 15.3 | 23.1 |  |  | 16.3 | 23.1 | |  |
| Daily or mostly | 26.3 | 20.2 |  |  | 11.9 | 20.2 | |  |
| Missing/unknown | 2.2 | 0.5 |  |  | 2.2 | 0.5 | |  |
| **Serologic variables*** |  |  |  |  |  |  | |  |
| Glucose (mmol/L) | 5.4±1.8 | 5.1±1.2 | 0.007 |  | 5.4±1.7 | 5.1±1.2 | | 0.04 |
| HbA1c (mmol/mol) | 37.6±7.4 | 36.1±6.8 | 0.02 |  | 38.4±9.2 | 36.1±6.8 | | 0.001 |
| CRP (mg/L) | 3.6±6.0 | 2.6±4.3 | 0.008 |  | 3.3 ±5.6 | 2.6±4.3 | | 0.009 |
| IGF-1 (nmol/L) | 21.5±5.9 | 21.4±5.7 | 0.92 |  | 19.8±5.1 | 21.4±5.7 | | 0.008 |
| SHBG (nmol/L) | 47.8±22.2 | 51.3±27.6 | 0.002 |  | 53.0±26.1 | 51.3±27.6 | | 0.57 |
| Testosterone (nmol/L) | 10.0±5.3 | 6.7±6.1 | <0.0001 |  | 8.6±6.1 | 6.7±6.1 | | 0.006 |
| Free testosterone (pmol/L) | 161±82.4 | 115±107 | <0.0001 |  | 128±93.1 | 115±107 | 0.32 | |

*Mean ± SD. †Median. Abbreviations: BMI, body mass index; CGC, cardia gastric cancer; CRP, C-reactive protein; HbA1c, glycated hemoglobin; IGF-1, insulin-like growth factor-1; NCGC, non-cardia gastric cancer; SD, standard deviation; SHBG, sex hormone-binding globulin

Supplementary table 5. Odds ratios and 95% confidence intervals for circulating hormones and non-cardia gastric cancer in men and women combined from the EPIC study

|  |  | |  | | **NCGC** | | |
| --- | --- | --- | --- | --- | --- | --- | --- |
|  |  | | **Cases/**  **Controls** | | **Crude OR^a^**  **(95% CI)** | | **Adjusted OR^b^**  **(95% CI)** |
|  |  | | **(172/172)** | |  | |  |
| **Insulin (pg/mL)^c^** |  | |  | |  | |  |
| <231 |  | | 27/29 | | Reference | | Reference |
| 231-323 |  | 14/22 | | 0.68 (0.29-1.61) | | 0.83 (0.27-2.52) | |
| >323 |  | 36/26 | | 1.48 (0.71-3.09) | | 1.91 (0.68-5.38) | |
| *P trend* |  | |  | | 0.20 | | 0.24 |
| Per 1-SD increase |  | |  | | 1.24 (0.89-1.72) | | 1.48 (0.91-2.39) |
| **C-peptide (pmol/L) ^d^** |  | |  | |  | |  |
| <531 |  | | 48/62 | | Reference | | Reference |
| 531-878 |  | | 57/52 | | 1.43 (0.84-2.44) | | 1.25 (0.68-2.31) |
| >878 |  | | 65/56 | | 1.59 (0.90-2.79) | | 1.66 (0.85-3.25) |
| *P trend* |  | |  | | 0.23 | | 0.33 |
| Per 1-SD increase |  | |  | | 1.41 (0.97-2.06) | | 1.08 (0.52-2.21) |
| **IGF-1 (ng/mL)** |  | |  | |  | |  |
| <74.4 |  | | 59/61 | | Reference | | Reference |
| 74.4-97.8 |  | | 62/59 | | 1.11 (0.63-1.95) | | 1.52 (0.79-2.91) |
| >97.8 |  | | 51/52 | | 1.02 (0.53-1.96) | | 1.47 (0.68-3.15) |
| *P trend* |  | |  | | 0.93 | | 0.42 |
| Per 1-SD increase |  | |  | | 1.04 (0.80-1.36) | | 1.25 (0.91-1.71) |
| **IGFBP-3 (ng/mL)** |  | |  | |  | |  |
| <2010 |  | | 69/55 | | Reference | | Reference |
| 2010-2338 |  | | 49/54 | | 0.69 (0.40-1.18) | | 0.71 (0.39-1.31) |
| >2338 |  | 54/63 | | 0.62 (0.35-1.09) | | 0.71 (0.37-1.37) | |
| *P trend* |  | |  | | 0.22 | | 0.48 |
| Per 1-SD increase |  | |  | | 0.68 (0.51-0.90) | | 0.70 (0.51-0.95) |
| **Adiponectin (ng/mL)** |  | |  | |  | |  |
| <5842 |  | | 67/61 | | Reference | | Reference |
| 5842-8634 |  | | 48/53 | | 0.81 (0.47-1.39) | | 0.82 (0.44-1.52) |
| >8634 |  | 57/58 | | 0.84 (0.44-1.62) | | 0.98 (0.45-2.14) | |
| *P trend* |  | |  | | 0.74 | | 0.76 |
| Per 1-SD increase |  | |  | | 0.87 (0.67-1.12) | | 0.97 (0.71-1.32) |
| **Leptin**  **(pg/mL)** |  | |  | |  | |  |
| <2695 |  | | 44/57 | | Reference | | Reference |
| 2695-6869 |  | | 60/54 | | 1.49 (0.83-2.69) | | 2.09 (1.02-4.31) |
| >6869 |  | | 67/60 | | 1.61 (0.85-3.03) | | 1.80 (0.77-4.23) |
| *P trend* |  | |  | | 0.25 | | 0.12 |
| Per 1-SD increase |  | |  | | 1.28 (0.97-1.70) | | 1.60 (1.06-2.42) |
| **Ghrelin**  **(pg/mL)** |  | |  | |  | |  |
| <176 |  | | 75/57 | | Reference | | Reference |
| 176-306 |  | | 57/58 | | 0.69 (0.40-1.21) | | 0.63 (0.33-1.19) |
| >306 |  | | 40/57 | | 0.46 (0.25-0.84) | | 0.42 (0.21-0.85) |
| *P trend* |  | |  | | 0.04 | | 0.05 |
| Per 1-SD increase |  | |  | | 0.69 (0.53-0.89) | | 0.66 (0.49-0.89) |

Sex-combined analyses was performed for hormones without sex-differences

^a^Crude model was integrally adjusted for the matching factors, including study center, sex, age at recruitment and date/time of blood collection

^b^Adjusted model was based on crude model with further adjustment for education level, smoking and body mass index

^c^For insulin, only fasting subjects were included

^d^Additionally adjusted for fasting status

All variables were log transformed

Not all of the cases/controls will sum to the total due to missing data

Abbreviations: CI, confidence interval; IGF-1, insulin-like growth factor-1; IGFBP-3, insulin-like growth factor-binding protein-3; NCGC, non-cardia gastric cancer; OR, odds ratio; SD, standard deviation

Supplementary table 6. Hazard ratios and 95% confidence intervals for circulating hormones and gastric cancer by subsite in men and women combined from the UK-Biobank study

|  |  | **CGC** | |  |  | **NCGC** | | |
| --- | --- | --- | --- | --- | --- | --- | --- | --- |
|  | **Cases** | **Crude HR^a^**  **(95% CI)** | **Adjusted HR^b^**  **(95% CI)** |  | **Cases** | **Crude HR^a^**  **(95% CI)** | | **Adjusted HR^b^**  **(95% CI)** |
|  | **(N=137)** |  |  |  | **(N=92)** |  | |  |
| **Glucose (mmol/L)** |  |  |  |  |  |  | |  |
| <4.7 | 38 | Reference | Reference |  | 21 | Reference | | Reference |
| 4.7-5.1 | 30 | 0.82 (0.49-1.36) | 0.83 (0.50-1.39) |  | 30 | 1.39 (0.78-2.50) | | 1.40 (0.78-2.51) |
| >5.1 | 49 | 1.09 (0.69-1.72) | 1.08 (0.67-1.72) |  | 30 | 1.26 (0.71-2.26) | | 1.27 (0.71-2.28) |
| *P trend* |  | 0.50 | 0.58 |  |  | 0.52 | | 0.52 |
| Per 1-SD increase |  | 1.14 (0.98-1.32) | 1.12 (0.96-1.30) |  |  | 1.11 (0.93-1.32) | | 1.12 (0.93-1.34) |
| **HbA1c (mmol/mol)** |  |  |  |  |  |  | |  |
| <33.6 | 35 | Reference | Reference |  | 24 | Reference | | Reference |
| 33.6-36.8 | 36 | 0.96 (0.59-1.59) | 0.89 (0.54-1.47) |  | 24 | 0.91 (0.50-1.64) | | 0.92 (0.51-1.66) |
| >36.8 | 56 | 1.13 (0.71-1.79) | 0.93 (0.58-1.50) |  | 41 | 1.14 (0.67-1.95) | | 1.13 (0.65-1.96) |
| *P trend* |  | 0.77 | 0.90 |  |  | 0.68 | | 0.73 |
| Per 1-SD increase |  | 1.08 (0.92-1.28) | 1.01 (0.85-1.20) |  |  | 1.15 (0.97-1.36) | | 1.15 (0.97-1.37) |
| **CRP (mg/L)** |  |  |  |  |  |  | |  |
| <0.8 | 31 | Reference | Reference |  | 20 | Reference | | Reference |
| 0.8-2.1 | 48 | 1.21 (0.75-1.97) | 1.02 (0.62-1.66) |  | 30 | 1.22 (0.68-2.20) | | 1.25 (0.69-2.27) |
| >2.1 | 51 | 1.42 (0.88-2.29) | 1.04 (0.62-1.72) |  | 38 | 1.51 (0.86-2.65) | 1.57 (0.86-2.87) | |
| *P trend* |  | 0.35 | 0.99 |  |  | 0.35 | | 0.33 |
| Per 1-SD increase |  | 1.19 (0.98-1.44) | 1.06 (0.86-1.30) |  |  | 1.13 (0.91-1.40) | | 1.14 (0.90-1.44) |
| **IGF-1 (nmol/L)** |  |  |  |  |  |  | |  |
| <18.9 | 40 | Reference | Reference |  | 37 | Reference | | Reference |
| 18.9-23.5 | 48 | 1.20 (0.77-1.89) | 1.29 (0.82-2.03) |  | 30 | 0.97 (0.59-1.60) | | 0.98 (0.59-1.61) |
| >23.5 | 41 | 1.21 (0.76-1.93) | 1.31 (0.81-2.09) |  | 21 | 0.80 (0.45-1.42) | | 0.81 (0.46-1.44) |
| *P trend* |  | 0.65 | 0.45 |  |  | 0.74 | | 0.75 |
| Per 1-SD increase |  | 1.10 (0.91-1.33) | 1.14 (0.95-1.38) |  |  | 0.88 (0.72-1.09) | | 0.89 (0.71-1.10) |

Sex-combined analyses was performed for hormones without sex-differences

^a^Crude model was stratified on age, sex, center, and town send deprivation index

^b^Adjusted model was based on crude model with further adjustment for education level, smoking, and body mass index

Not all of the cases will sum to the total due to missing data

Abbreviations: CGC, cardia gastric cancer; CI, confidence interval; CRP, C-reactive protein; HbA1c, glycated hemoglobin; HR, hazard ratio; IGF-1, insulin-like growth factor-1; NCGC, non-cardia gastric cancer; SD, standard deviation

Supplementary table 7. Pearson correlations between hormones in EPIC study

|  | **Insulin** | **C-peptide** | **IGF-1** | **IGFBP-3** | **Adiponectin** | **Leptin** | **Ghrelin** | **Androstenedione** | **DHEA** | **Estrone** | **Estradiol** | **SHBG** | **Testosterone** | **Progesterone** | **Free testosterone** | **Free estradiol** |
| --- | --- | --- | --- | --- | --- | --- | --- | --- | --- | --- | --- | --- | --- | --- | --- | --- |
| **Insulin** | 1.00 | 0.501 | 0.069 | 0.019 | -0.252 | 0.181 | -0.148 | -0.158 | -0.082 | -0.030 | 0.033 | -0.102 | 0.031 | -0.219 | 0.043 | 0.049 |
| **C-peptide** |  | 1.00 | 0.061 | -0.021 | -0.149 | 0.083 | -0.098 | -0.087 | -0.068 | -0.003 | 0.022 | -0.058 | 0.020 | -0.147 | 0.023 | 0.028 |
| **IGF-1** |  |  | 1.00 | 0.138 | -0.182 | -0.209 | -0.120 | 0.296 | 0.207 | 0.224 | 0.299 | -0.102 | 0.386 | 0.229 | 0.382 | 0.303 |
| **IGFBP-3** |  |  |  | 1.00 | 0.172 | 0.130 | 0.080 | -0.081 | 0.040 | -0.055 | -0.181 | 0.076 | -0.259 | -0.088 | -0.255 | -0.182 |
| **Adiponectin** |  |  |  |  | 1.00 | 0.158 | 0.129 | -0.301 | -0.142 | -0.270 | -0.408 | 0.437 | -0.508 | -0.139 | -0.554 | -0.466 |
| **Leptin** |  |  |  |  |  | 1.00 | -0.011 | -0.360 | -0.223 | -0.164 | -0.373 | 0.061 | -0.570 | -0.318 | -0.549 | -0.363 |
| **Ghrelin** |  |  |  |  |  |  | 1.00 | -0.025 | 0.021 | -0.058 | -0.106 | 0.023 | -0.168 | -0.060 | -0.167 | -0.111 |
| **Androstenedione** |  |  |  |  |  |  |  | 1.00 | 0.707 | 0.595 | 0.438 | -0.162 | 0.531 | 0.595 | 0.528 | 0.446 |
| **DHEA** |  |  |  |  |  |  |  |  | 1.00 | 0.331 | 0.142 | -0.094 | 0.210 | 0.375 | 0.213 | 0.151 |
| **Estrone** |  |  |  |  |  |  |  |  |  | 1.00 | 0.756 | -0.107 | 0.525 | 0.407 | 0.512 | 0.741 |
| **Estradiol** |  |  |  |  |  |  |  |  |  |  | 1.00 | -0.185 | 0.787 | 0.465 | 0.773 | 0.987 |
| **SHBG** |  |  |  |  |  |  |  |  |  |  |  | 1.00 | -0.239 | -0.053 | -0.387 | -0.340 |
| **Testosterone** |  |  |  |  |  |  |  |  |  |  |  |  | 1.00 | 0.404 | 0.987 | 0.797 |
| **Progesterone** |  |  |  |  |  |  |  |  |  |  |  |  |  | 1.00 | 0.390 | 0.452 |
| **Free testosterone** |  |  |  |  |  |  |  |  |  |  |  |  |  |  | 1.00 | 0.808 |
| **Free estradiol** |  |  |  |  |  |  |  |  |  |  |  |  |  |  |  | 1.00 |

Abbreviations: DHEA, dehydroepiandrosterone; IGF-1, insulin-like growth factor-1; IGFBP-3, insulin-like growth factor-binding protein-3; SHBG, sex hormone-binding globulin

Supplementary table 8. Pearson correlations between hormones in ATBC study

|  | **Insulin** | **IGF-1** | **IGFBP-3** | **Adiponectin** | **Leptin** | **Ghrelin** | **Androstenedione** | **Androsterone** | **DHEA** | **Estrone** | **Estradiol** | **SHBG** | **Testosterone** | **Dihydrotestosterone** | **Progesterone** | **Free estradiol** | **Free testosterone** |
| --- | --- | --- | --- | --- | --- | --- | --- | --- | --- | --- | --- | --- | --- | --- | --- | --- | --- |
| **Insulin** | 1.00 | 0.035 | -0.071 | -0.444 | 0.567 | -0.070 | -0.178 | -0.233 | -0.133 | -0.028 | -0.073 | -0.320 | -0.317 | -0.338 | -0.163 | 0.082 | -0.155 |
| **IGF-1** |  | 1.00 | 0.638 | 0.023 | 0.046 | 0.034 | 0.059 | 0.235 | 0.072 | -0.073 | -0.085 | -0.163 | -0.034 | -0.023 | -0.014 | 0.004 | 0.112 |
| **IGFBP-3** |  |  | 1.00 | -0.009 | 0.095 | 0.024 | -0.006 | 0.187 | 0.099 | 0.015 | -0.024 | -0.228 | -0.082 | -0.056 | 0.124 | 0.100 | 0.104 |
| **Adiponectin** |  |  |  | 1.00 | -0.341 | 0.101 | 0.198 | 0.049 | 0.109 | 0.014 | 0.099 | 0.466 | 0.303 | 0.376 | 0.098 | -0.139 | -0.001 |
| **Leptin** |  |  |  |  | 1.00 | -0.001 | -0.237 | -0.139 | -0.111 | 0.015 | -0.037 | -0.391 | -0.411 | -0.418 | -0.264 | 0.170 | -0.190 |
| **Ghrelin** |  |  |  |  |  | 1.00 | -0.056 | -0.094 | -0.043 | -0.008 | 0.001 | -0.059 | -0.032 | -0.062 | -0.038 | 0.027 | 0.014 |
| **Androstenedione** |  |  |  |  |  |  | 1.00 | 0.481 | 0.617 | 0.448 | 0.394 | 0.189 | 0.469 | 0.424 | 0.469 | 0.298 | 0.446 |
| **Androsterone** |  |  |  |  |  |  |  | 1.00 | 0.632 | 0.320 | 0.199 | -0.087 | 0.228 | 0.312 | . | 0.253 | 0.368 |
| **DHEA** |  |  |  |  |  |  |  |  | 1.00 | 0.304 | 0.228 | 0.078 | 0.195 | 0.182 | 0.314 | 0.204 | 0.221 |
| **Estrone** |  |  |  |  |  |  |  |  |  | 1.00 | 0.635 | 0.021 | 0.176 | 0.132 | 0.328 | 0.619 | 0.210 |
| **Estradiol** |  |  |  |  |  |  |  |  |  |  | 1.00 | 0.260 | 0.595 | 0.473 | 0.468 | 0.862 | 0.517 |
| **SHBG** |  |  |  |  |  |  |  |  |  |  |  | 1.00 | 0.614 | 0.667 | 0.159 | -0.258 | -0.056 |
| **Testosterone** |  |  |  |  |  |  |  |  |  |  |  |  | 1.00 | 0.859 | 0.415 | 0.283 | 0.750 |
| **Dihydrotestosterone** |  |  |  |  |  |  |  |  |  |  |  |  |  | 1.00 | . | 0.099 | 0.539 |
| **Progesterone** |  |  |  |  |  |  |  |  |  |  |  |  |  |  | 1.00 | 0.404 | 0.378 |
| **Free estradiol** |  |  |  |  |  |  |  |  |  |  |  |  |  |  |  | 1.00 | 0.558 |
| **Free testosterone** |  |  |  |  |  |  |  |  |  |  |  |  |  |  |  |  | 1.00 |

Abbreviations: DHEA, dehydroepiandrosterone; IGF-1, insulin-like growth factor-1; IGFBP-3, insulin-like growth factor-binding protein-3; SHBG, sex hormone-binding globulin

Supplementary table 9. Pearson correlations between hormones in UK-Biobank study

|  | **Glucose** | **HbA1c** | **CRP** | **IGF-1** | **SHBG** | **Testosterone** | **Free testosterone** |
| --- | --- | --- | --- | --- | --- | --- | --- |
| **Glucose** | 1.00 | 0.494 | 0.085 | -0.070 | -0.142 | -0.061 | 0.024 |
| **HbA1c** |  | 1.00 | 0.171 | -0.076 | -0.227 | -0.074 | 0.065 |
| **CRP** |  |  | 1.00 | -0.214 | -0.256 | -0.052 | 0.107 |
| **IGF-1** |  |  |  | 1.00 | -0.122 | 0.027 | 0.108 |
| **SHBG** |  |  |  |  | 1.00 | 0.209 | -0.420 |
| **Testosterone** |  |  |  |  |  | 1.00 | 0.789 |
| **Free testosterone** |  |  |  |  |  |  | 1.00 |

Abbreviations: CRP, C-reactive protein; HbA1c, glycated hemoglobin; IGF-1, insulin-like growth factor-1; SHBG, sex hormone-binding globulin
